# Genome-wide discovery of structured noncoding RNAs in bacteria

**DOI:** 10.1186/s12866-019-1433-7

**Published:** 2019-03-22

**Authors:** Shira Stav, Ruben M. Atilho, Gayan Mirihana Arachchilage, Giahoa Nguyen, Gadareth Higgs, Ronald R. Breaker

**Affiliations:** 1 Department of Molecular, Cellular and Developmental Biology, Yale University, New Haven, USA; 2 Department of Molecular Biophysics and Biochemistry, Yale University, New Haven, USA; 3 Howard Hughes Medical Institute, Yale University, New Haven, CT 06520 USA

## Abstract

**Background:**

Structured noncoding RNAs (ncRNAs) play essential roles in many biological processes such as gene regulation, signaling, RNA processing, and protein synthesis. Among the most common groups of ncRNAs in bacteria are riboswitches. These *cis*-regulatory, metabolite-binding RNAs are present in many species where they regulate various metabolic and signaling pathways. Collectively, there are likely to be hundreds of novel riboswitch classes that remain hidden in the bacterial genomes that have already been sequenced, and potentially thousands of classes distributed among various other species in the biosphere. The vast majority of these undiscovered classes are proposed to be exceedingly rare, and so current bioinformatics search techniques are reaching their limits for differentiating between true riboswitch candidates and false positives.

**Results:**

Herein, we exploit a computational search pipeline that can efficiently identify intergenic regions most likely to encode structured ncRNAs. Application of this method to five bacterial genomes yielded nearly 70 novel genetic elements including 30 novel candidate ncRNA motifs. Among the riboswitch candidates identified is an RNA motif involved in the regulation of thiamin biosynthesis.

**Conclusions:**

Analysis of other genomes will undoubtedly lead to the discovery of many additional novel structured ncRNAs, and provide insight into the range of riboswitches and other kinds of ncRNAs remaining to be discovered in bacteria and archaea.

**Electronic supplementary material:**

The online version of this article (10.1186/s12866-019-1433-7) contains supplementary material, which is available to authorized users.

## Background

Ongoing efforts to discover and characterize noncoding RNAs (ncRNAs) in bacteria and archaea are expanding our understanding of gene regulation and are revealing new aspects of biology. In the area of riboswitches [1–3], computational search strategies have become the most productive means to uncover new candidates. For example, several reports [4–6] describe the use of computational approaches to find multiple novel riboswitch classes by comparative sequence analyses [7] of the growing collection of bacterial genomic sequence data. Computational discovery approaches, along with more traditional bacterial genetics methods, have led to the experimental validation of over 40 distinct riboswitch classes that respond to small metabolite or ion ligands [8].

Data derived from these initial finds has been used to support the prediction that perhaps thousands of additional riboswitch classes await detection [1, 8–10]. The discovery of large numbers of additional riboswitch classes would expand the known diversity of RNA structures that selectively recognize fundamental biological ligands [3, 8]. Such findings would also increase opportunities to observe riboswitches that use sophisticated architectures to bind ligands cooperatively [11–14], to function as two-input genetic logic gates [15, 16], or to regulate the action of ribozymes [16, 17]. Moreover, experimental validation of new riboswitch classes can reveal hidden aspects of biology. For example, numerous fluoride toxicity resistance genes were first revealed by the discovery of fluoride-responsive riboswitches [18, 19]. Similarly, numerous guanidine toxicity resistance genes were initially uncovered by establishing the existence of a series of guanidine-sensing riboswitches [13, 20, 21].

These intriguing prospects have motivated us to create a computational search pipeline that can be used to efficiently discover many additional structured ncRNA candidates, including rare riboswitch classes. Current computational methods have proven very effective for identifying common, well-structured ncRNAs, including riboswitches [8], ribozymes [22], and protein-binding RNA motifs [23]. Unfortunately, more recently discovered riboswitch classes tend to be rarer than those discovered earlier [8, 9]. Riboswitches and other structured ncRNAs are progressively becoming more difficult to separate from ncRNAs that typically have less secondary structure. Thus, it is becoming more problematic to distinguish ncRNAs from false positives that are due to the ‘noise’ resulting from chance sequence and structure similarities between unrelated sequences. Also, improvements in computational search algorithms are become especially important as more genomic and metagenomic sequence data becomes available, causing search demands to grow exponentially.

Almost without exception, riboswitches and other structured ncRNAs reside outside of protein-coding regions. Thus, DNA databases to be searched can be substantially reduced in size by examining only the noncoding regions of each genome. To further reduce the amount of DNA sequence data to be searched, we sought to focus only on the intergenic regions (IGRs) that have the properties consistent with serving as a template for the production of structured ncRNAs. Riboswitches and other structured ncRNAs often reside in relatively long IGRs that typically exhibit a roughly equal distribution of all four nucleotide types, reflecting their need to form complex and stable secondary and tertiary structures. For example, structured ncRNAs tend to have a high percentage of G and C (guanosine and cytidine) nucleotides compared to other regions of the genomes of organisms that are naturally biased in favor of A and T (adenosine and thymidine) nucleotides [24–27]. Organisms whose genomes are AT-rich and also have mostly short IGRs are particularly attractive for bioinformatic analyses that pre-sort DNA sequence data based on these two parameters [28].

Computational searches for GC-rich segments of otherwise AT-rich genomes initially were reported in 2002, and revealed novel ncRNA candidates in bacterial [24] and archaeal [25] species. However, the amount of microbial sequence data available at that time precluded more extensive comparative sequence analyses to establish consensus sequence and structural models typical of complex-folded RNA motifs. In a study published in 2009, we sorted the IGRs of *Pelagibacter ubique* (*P. ubique*) based on both length and GC content to identify four novel structured ncRNA motifs [27], including a novel riboswitch type that selectively responds to the coenzyme *S*-adenosylmethionine [28]. The outcomes of this general analytical approach, hereafter termed ‘GC-IGR’ analysis, demonstrate that even rare structured noncoding RNAs can be uncovered. Once each candidate ncRNA is identified, structural models and hypotheses regarding biochemical functions can be more readily generated, particularly by subsequently using comparative sequence analysis to expand the number of natural representatives of each motif.

In the current report, we present a robust method that expands the GC-IGR search approach that can be used to examine large numbers of bacterial genomes. Specifically, we initially examined the IGRs from 2807 fully sequenced bacterial and archaeal genomes available in RefSeq [29] release 63. We determined that 1200 genomes were suitable for further investigation, and comprehensively analyzed collections of IGRs of particular interest from five genomes by employing a multistep process to ensure consistent evaluation of each IGR’s possible functions. Novel elements discovered in a given genome can then be both forward- and back-annotated to all bacterial genomes undergoing analysis, thereby reducing the number of IGRs with unknown genetic contents (hereafter called ‘unknown IGRs’) that remain to be assessed in other genomes.

**Fig. 1:**
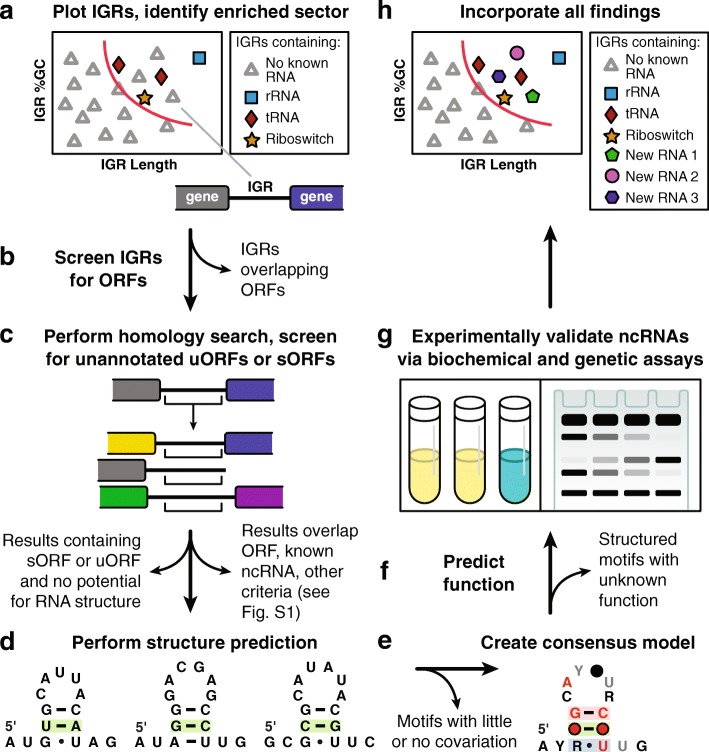
Overview of the search pipeline. Schematic representation of the GC-IGR analysis workflow. **a-h** Annotations in bold text represent major steps in the analytical pipeline. For a more detailed version of the pipeline, see Additional file 2: Figure S1

The detailed findings from five bacterial genomes whose IGRs have been fully analyzed by this GC-IGR computational pipeline (Fig. 1) are described herein. These search efforts have uncovered many novel structured ncRNA candidates and peptide-encoding short open reading frames (ORFs) (information on all novel findings is provided in Additional file 1: Table S1). Particularly noteworthy is the discovery of a candidate riboswitch class that responds to an intermediate in the biosynthetic pathway for the coenzyme thiamin pyrophosphate (TPP). This GC-IGR search approach can now be used to efficiently uncover the vast majority of structured ncRNAs encoded by the genomes of hundreds of bacterial species.

## Results and discussion

### Choosing genomes for complete analysis by the GC-IGR bioinformatics pipeline

GC-IGR analyses were initiated by first examining the properties of 2807 bacterial genomes. To scout for suitable genomes, we generated 2D plots depicting all putative IGRs from a given genome, wherein each IGR was sorted based on two parameters: its length in nucleotides (X axis) and its %GC content (Y axis) (Fig. 1a). Plots also include the length and %GC values for the structured ncRNAs already known to be carried by the genome under examination. These known ncRNAs included tRNAs, riboswitches, and other functional RNAs such as the signal recognition particle (SRP) RNA [30], RNase P [31], 6S [32] and tmRNA [33].

This scouting exercise revealed two characteristics that are important for our search strategy to be most productive: (i) distinctive IGR characteristics and (ii) an abundance of known ncRNA classes. Regarding this first characteristic, the distributions of IGRs vary greatly among different bacterial and archaeal species when sorted by length and %GC content. %GC values for the genomes originally assessed ranged from 10.0 to 79.4 (average of 43.5), and the average IGR lengths for each genome in nucleotides ranged from 59.9 to 1110.8 (overall average of 172.8). Genomes preferred for in-depth analysis should have very low %GC content and very short average IGR lengths. These characteristics favor robust separation of the more GC-rich and longer IGRs carrying novel ncRNAs from those that likely do not. For the current study, we also favored the analysis of genomes with fewer IGRs than typical (overall average of 2407.9 annotated IGRs per genome) to further reduce the number of unknown IGRs that required detailed assessments.

Regarding the second characteristic, some bacterial species carry very few known classes of structured ncRNA motifs, whereas others have abundant riboswitches and other types of ncRNAs in addition to their typical complement of tRNA and ribosomal RNA genes. We speculate that genomes already found to carry a diversity of structured ncRNA classes are likely to have more undiscovered classes compared to those genomes that are mostly devoid of currently known RNA classes.

All genomes were ranked by their average IGR length, their average IGR GC content, and lastly by the number of IGRs per genome. Genomes on the lower end of all three IGR properties that also contained a diverse set of ncRNAs, particularly structured regulatory RNAs, formed the collection of 1200 genomes from which we chose those for further analysis. These genomes tend to have an abundance of ncRNA classes that cluster in one sector of the plot, away from the majority of unknown IGRs. Thus, unknown IGRs that cluster in this same sector are more likely to carry novel ncRNA motifs. Finally, we also considered phylogenetic diversity when making our genome choices, and ultimately five (out of many suitable genomes) were analyzed in detail for the current study: Alphaproteobacterium HIMB5 (NC_018643.1), *Clostridium novyi* NT (NC_008593.1), *Thermovirga lienii* (NC_016148.1), *Arcobacter* sp. L (NC_017192.1), and *Baumannia cicadellinicola* (NC_007984.1).

### Defining the boundaries of the sector containing IGRs to be further analyzed

For each genome analyzed in detail, the noncoding IGRs as defined by existing genome annotations (RefSeq assembly files [29]) were plotted (Fig. 1a). The known ncRNAs are identified by a colored symbol and unknown IGRs are represented with gray triangles. A boundary was then created to define which IGRs would become targets for further analysis. The placement of the boundary is achieved manually, but guided by our desire to position most of the known ncRNAs to the right and above the boundary without including so many unknown IGRs that detailed analysis becomes impractical. Generally, the ratio of known ncRNAs to unknown IGRs is close to 1 in the sector chosen for further analysis.

### Analysis of unknown IGRs from alpha proteobacterium HIMB5 uncovers a diversity of novel genetic elements

Unknown IGRs residing within the chosen sector were subsequently analyzed with a semi-automated computational pipeline (Fig. 1b-f) that builds upon previous search algorithms used to discover novel ncRNAs [5, 6] or to further analyze known ncRNAs [8]. For additional details of the computational analyses, see the Methods section and Additional file 2: Figure S1. If IGRs are found to carry a novel candidate structured ncRNA, then additional efforts can be pursued to experimentally determine its biochemical and biological function (Fig. 1g). Newly discovered ncRNA motifs can then be annotated throughout the genomes that carry them, and the 2D genomic plots updated accordingly (Fig. 1h).

**Fig. 2:**
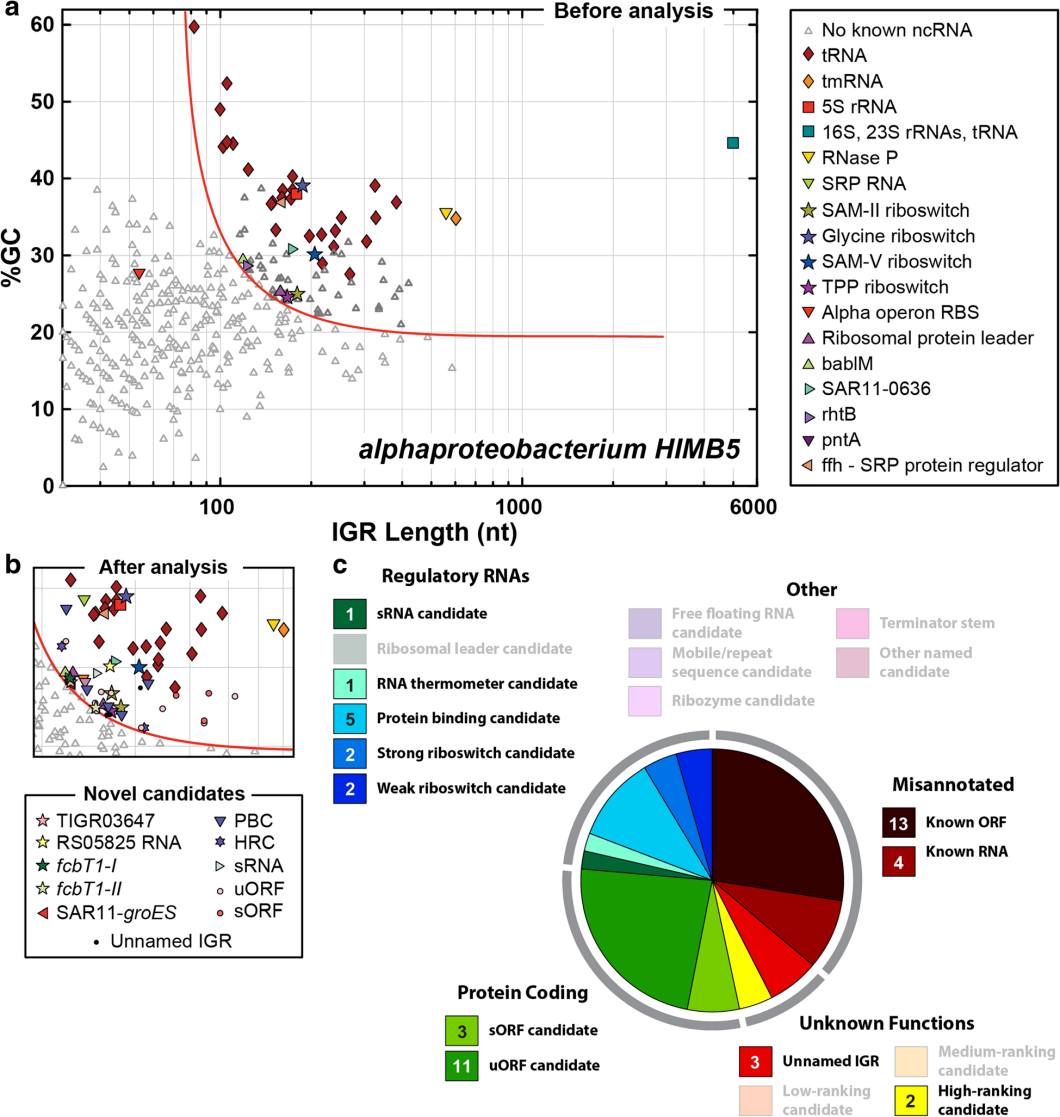
Plots of the IGRs from the HIMB5 genome sorted based on IGR length and GC content. **a** IGR plot prior to conducting our detailed analyses. Red line represents the boundary between unknown IGRs chosen for further analysis (upper right) and those that were not chosen (lower left). IGRs selected for further analysis are depicted with dark gray triangles whereas those not chosen are depicted with light gray triangles. **b,** (Top) A portion of the sector of interest from the HIMB5 IGR plot after analysis, updated to remove false IGRs that overlap known ORFs, to annotate IGRs carrying previously known ncRNAs, and to include all novel motifs identified in this study. No changes in the plot occurred outside of the area depicted. (Bottom) List of novel motif candidates. **c,** Summary of the classification of all 47 unknown IGRs from HIMB5 chosen for further analysis. Classifications are organized into five main groups (gray arcs) as annotated, wherein “unknown functions” encompasses categories 1 through 4 (unnamed, LRC, MRC and HRC), and the remaining groups are derived from category 5 (named), as described in the main text. The number of novel examples classified in each group are provided in the colored boxes. Classifications depicted as partially transparent lack a representative in the sector of interest in this genome. See Additional file 1: Table S1 for additional details regarding novel motifs

For this study, the first genome to be fully analyzed by the GC-IGR approach was from alpha proteobacterium HIMB5 (hereafter referred to as HIMB5). This bacterial species is a member of the same SAR11 clade as *P. ubique*, which we previously analyzed using an earlier version of the GC-IGR search pipeline [27]. Both organisms have similar IGR properties that are favorable for our analytical approach, including relatively few IGRs, which are mostly short (~ 100 nucleotides on average), and a remarkably low (~ 25%) genomic GC content. The GC-IGR genome plot for HIMB5 (Fig. 2a) reveals that there is excellent separation of the known ncRNAs from the vast majority of the unknown IGRs.

A total of 47 unknown IGRs reside in the chosen sector, of which 13 were determined via our computational pipeline (Additional file 2: Figure S1) to actually represent coding regions for known proteins that were not annotated in the original DNA sequence dataset. Similarly, four of the remaining 34 unknown IGRs were found to actually represent known RNAs that were also not annotated in the original dataset. These RNAs include a tRNA, a SAM-II riboswitch, SRP RNA, and an *rpsB* RNA (a ribosomal protein leader uncovered by our previous GC-IGR study with *P. ubique*)^27^. Rediscovery of known ncRNAs that were unannotated in the original genome dataset demonstrate that our computational pipeline is well suited to uncover structured ncRNAs.

After removing these coding regions and previously known ncRNAs, 30 unknown IGRs remained. Each unknown IGR was then carefully examined by comparative sequence analysis to identify additional representatives that conform to the same motif class and to establish a consensus sequence and secondary structure model. All 30 unknown IGRs have subsequently been classified in a manner that reflects either its predicted function or its potential relevance as a structured nucleic acid. Our naming system groups IGRs into one of five general categories:

1. Unnamed: Insufficient evidence to classify.
2. Low-ranking candidate (LRC): Typically fewer than 5 unique representatives and a poor consensus model.
3. Medium-ranking candidate (MRC): Typically fewer than 20 unique representatives and/or a poor consensus model.
4. High-ranking candidate (HRC): Many representatives and a good consensus model, but insufficient information regarding possible function.
5. Named candidate: Could be rare, but usually has many representatives with a good consensus model and some evidence supporting a hypothesis for function.

A summary of our classification of the 30 unknown IGRs from HIMB5 is presented in Additional file 1: Table S1. An updated IGR genome plot incorporating all the new classifications, as well as the removal of IGRs overlapping known protein coding regions, is presented in Fig. 2b, and the total diversity of findings for all IGRs analyzed is presented in graphical form in Fig. 2c. Notable findings include 11 different upstream open reading frames [34, 35] (uORFs) wherein each is predicted to regulate expression of the main ORF located immediately downstream. At least three additional short open reading frames [36, 37] (sORFs) appear to be present, which presumably produce short peptides that have as yet undefined biological functions. RNA and DNA structures that bind protein factors to control gene expression are also likely to be common in bacteria [38–40], and therefore it is not surprising that we identified five candidates predicted to be bound by transcription factors or other proteins.

Intriguingly, we also identified four structured ncRNA domains that we predict might function as ligand-binding riboswitches that regulate the expression of downstream ORFs. Each of these riboswitch candidates is described in greater detail below. For the novel motifs identified in the HIMB5 genome, all are phylogenetically confined to the SAR11 clade or metagenomic DNA sequences that have not been assigned to specific species. This is consistent with our hypothesis that many riboswitch classes exist, but that they are likely to be rarer than most classes reported previously [8]. Regardless, these novel ncRNA candidate classes, along with various other predicted motifs and ORFs, can now be added to our future GC-IGR plots so that they are not continually rediscovered when analyzing additional genomes.

### Novel riboswitch candidates from the HIMB5 genome

**Fig. 3:**
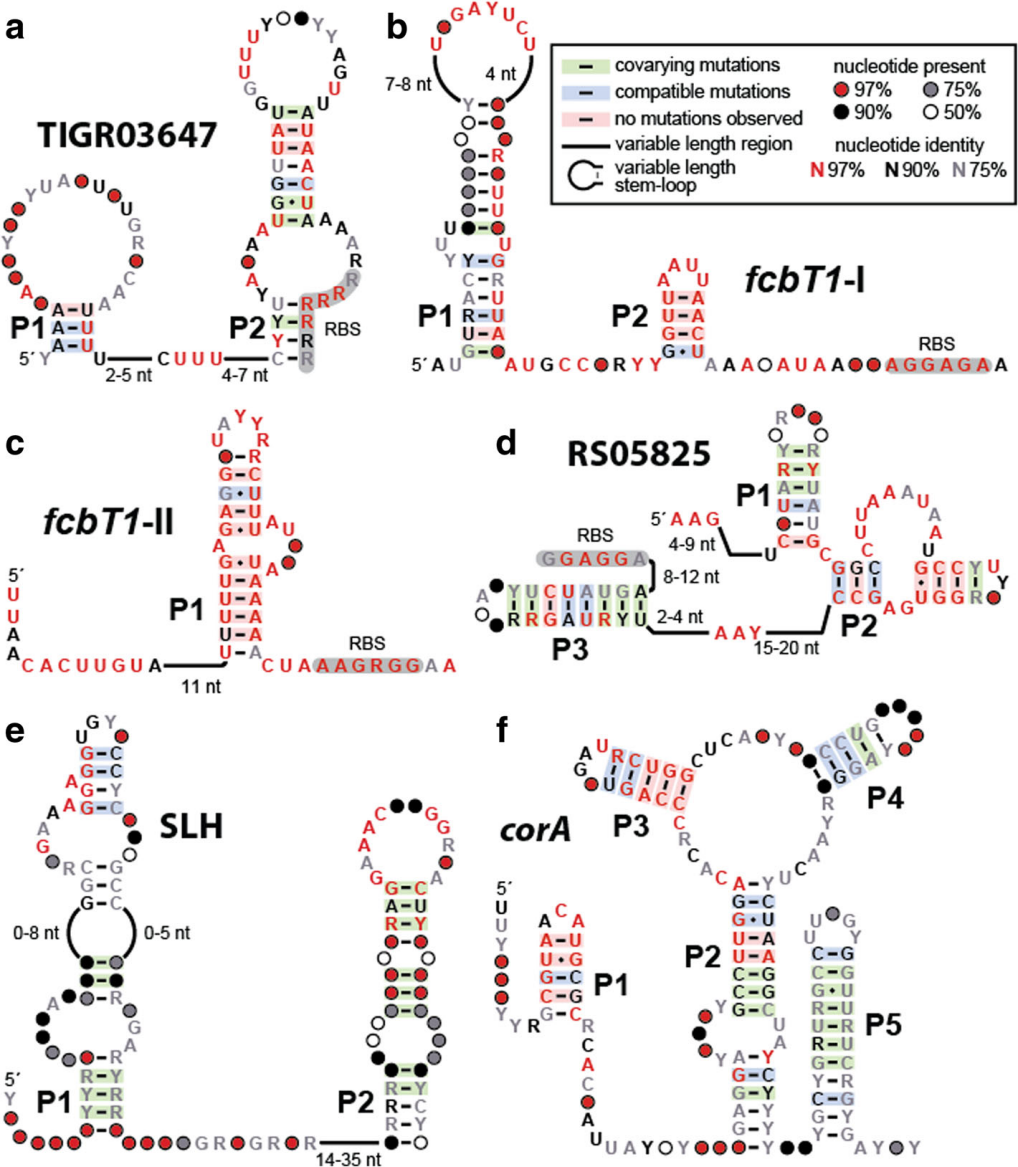
Sequence and secondary structure models for several candidate riboswitch classes identified in this study. **a-d** Four candidate riboswitch classes present in the HIMB5 genome. **e** A candidate riboswitch class present in the genome of *T. lienii*. **f** A candidate riboswitch class present in the genome of *B. cicadellinicola*. These consensus models are created by comparing all unique sequence representatives for each motif that were uncovered by homology searches of RefSeq 76 and certain metagenomic databases. See the text for details regarding each of these motifs and for hypotheses regarding their biological functions. Note that another riboswitch candidate, *thiS*, identified in the genome of *C. novyi* is presented in Fig. 4a

We anticipate that riboswitches will be a major type of structured ncRNA class encountered in our searches. Indeed, we have uncovered four novel ncRNA motifs in the HIMB5 genome, and three from the other genomes, that represent reasonable candidates for riboswitch function (Fig. 3). The HIMB5 riboswitch candidates are described below, along with hypotheses regarding the ligands they possibly sense. Note that each distinct candidate riboswitch class is typically named after the gene with which it is most commonly associated. As the ligands for riboswitch candidates are eventually experimentally validated, these original names are replaced to best describe the natural targets for the RNA aptamers.

These examples also highlight the various factors that can be considered when evaluating the possible functions of the structured ncRNAs uncovered by computational search methods. Large numbers of unique representatives that are well-conserved among diverse organisms suggest the sequence and structure of the ncRNA is critical for an important biological function. The abundance of well-conserved nucleotides and base-paired substructures also suggests the motif has a biochemical function that demands architectural sophistication. Finally, the proximity to gene control segments such as intrinsic terminator stems [41] or ribosome binding sites (RBS or Shine-Dalgarno sequence) [42] suggests a *cis-*regulatory role, wherein the ncRNA motif is transcribed as part of the mRNA it regulates. However, assessing the accuracy of such predictions cannot easily be achieved without experimentally testing any hypotheses regarding function. Therefore our assignments for these motifs as riboswitch candidates are necessarily highly subjective and tenuous. Thus, hypotheses regarding possible functions of these RNAs might require adjustments as additional information becomes available.

### *The* TIGR03647 *riboswitch candidate*

The *TIGR03647* riboswitch candidate (Fig. 3a) has 770 unique examples. The architecture of this motif includes a base-paired substructure (P2) that encompasses the predicted RBS, strongly suggesting that the motif functions as a *cis-*acting regulatory element to control gene expression at the level of translation. Similarly, the conserved CUUU sequence that is flanked by the two hairpin substructures also might base-pair with the purine-rich RBS sequence to block translation initiation. The absence of evidence for a uORF suggests that the RNAs might directly bind a small ligand to change gene expression, but other plausible hypotheses other than riboswitch function include binding by a protein or RNA regulatory factor.

The *TIGR03647* motif is always located immediately upstream of a gene coding for the small subunit of a putative solute/sodium symporter. This first gene is almost always followed immediately by the gene for the large subunit (*TIGR03648*) for this transporter complex. In our experience, the annotations for transporter genes are commonly inaccurate [18, 20], and in this instance it is also not known what partner ligand might be transported along with the ion. Therefore it is difficult to propose a compelling hypothesis for the natural ligand sensed by the putative riboswitch and the transporter whose production it controls. Four potential *cis-*regulatory RNA motifs have previously [43] been found upstream of ORFs encoding proteins predicted to be associated with sodium, but none are similar to the *TIGR03647* motif, either in structure or phylogenetic distribution.

### *The* fcbT1*-I and* fcbT1*-II motifs*

Both the *fcbT1*-I (Fig. 3b) and *fcbT1*-II (Fig. 3c) motif RNAs are uncommon (28 and 19 unique examples, respectively), which makes speculation of their possible functions exceedingly difficult. Both are located upstream of the gene that encodes FcbT1, which is a TRAP-type mannitol/chloroaromatic compound transport system involved in the uptake of C4-dicarboxylates [44]. Also due to the small number of representatives for these candidates, it is difficult to determine the true importance of the conserved nucleotides, and to predict secondary structure features with confidence. Currently, the proposed structures are exceedingly simple, which is not typical of most known riboswitch classes. However, given their proximity to the RBS and start codon of the *fcbT1* gene, these motifs appear to be *cis*-regulatory candidates. Although it seems possible that the *fcbT1*-I and *fcbT1*-II motifs could function as metabolite-binding riboswitches, we consider them to be weak candidates compared to other candidates identified in this study.

### *The* RS05825 *riboswitch candidate*

The RS05825 motif (Fig. 3d) has 80 unique examples and is always found upstream of a gene coding for a protein of unknown function (HIMB5_RS05825). The predicted secondary structure of this motif includes three hairpin substructures wherein P2 carries a well-conserved asymmetric internal loop. The P3 stem resides 8 to 12 nucleotides upstream of the predicted RBS for the associated ORF. This architecture strongly suggests that the *RS05825* motif is a *cis-*regulatory element that controls gene expression at the level of translation. However, without clarity on the function of the resulting protein, we cannot formulate a compelling hypothesis for the possible riboswitch ligand at this time.

### Summary of the outcomes for the analysis of 47 unknown IGRs from HIMB5

The completion of our analysis of the HIMB5 genome provides an initial view of the types and quantities of candidate ncRNA motifs and other biological systems that might remain to be discovered in other bacterial genomes. We organized our findings into five general categories (Fig. 2c). Approximately one-third of the 47 unknown IGRs actually correspond to known protein-coding genes that were not properly annotated in the original genomic sequence dataset. Approximately one quarter of the IGRs carry short protein-coding regions that appear to function either as regulatory uORFs [34, 35] or as sORFs [36, 37] whose tiny peptide products have unknown biological functions (see Additional file 1: Table S1 and Additional file 3: Supplementary text for details on other motifs not featured in the main text).

We cannot be certain about the predicted functions for any newly-found motif without experimental evidence for our hypotheses. However, we speculate that approximately a dozen of the IGRs analyzed (including the four candidate riboswitches) likely function as regulatory RNA structures. For example, we identified five novel protein-binding candidates and a possible RNA thermometer [45]. It is important to note that some protein-binding candidates might actually function at the level of single-stranded DNA, which might be the case for motifs associated with genes for DNA-binding proteins (see Additional file 1: Table S1 and Additional file 3: Supplementary text for additional details).

In contrast, we only identified a single small RNA (sRNA) that presumably regulates gene expression by forming base-pairing interactions with target mRNAs [38, 39]. Typically, sRNAs do not form extensive self-structure, and therefore should only rarely reside in the sector we examined to discover structured ncRNAs. Thus, sRNAs are likely to be largely missed by our search method even if they are common in the species under examination.

Despite the frequency of rediscovering known protein-coding regions and the discovery of additional protein-coding uORFs and sORFs, this initial dataset indicates that there are plenty of novel discoveries of ncRNAs and other genetic elements to be made among bacterial genomes. However, among the HIMB5 IGRs examined, four carry possible ncRNA motifs for which we could not provide reasonable hypotheses regarding function (HRC-1 and HRC-2) or determine structure-forming potential with confidence (Unnamed IGRs) (Fig. 2c). Thus, our search method can generate a collection of sequences that are enriched for functional ncRNA motifs, but additional studies will be needed to determine if individual representatives of each motif have complex biological functions.

### Analysis of four additional bacterial genomes exposes many novel nucleic acid motifs and a unique riboswitch candidate

The GC-IGR analysis methods demonstrated for the HIMB5 genome are readily applied to other genomes that have similar characteristics. For the current study, we chose to examine four additional genomes to prepare for a potentially larger campaign to analyze hundreds of bacterial genomes in search of novel structured ncRNA domains. Our objectives were to identify methodology weaknesses that would otherwise preclude large-scale analysis of genomes, and to gain a larger perspective on the prospects for making future discoveries. Below are summaries of our findings from these four genomes.

*The* Clostridium novyi *genome. C. novyi*, a member of the Firmicutes phylum, was chosen for analysis because it is rich in known classes of structured regulatory RNAs and possesses a well-defined clustering of the IGRs containing these RNAs (Additional file 4: Figure S2). *C. novyi* is a disease-causing organism classified in the same genus as numerous other pathogenic bacteria, including *Clostridium botulinum*, *Clostridium difficile*, *Clostridium perfringens*, *Clostridium sordellii*, and *Clostridium tetani* [46]. The poor IGR properties of these latter genomes make them much more difficult to analyze using our GC-IGR pipeline, but we reasoned that novel RNAs discovered in *C. novyi* are likely to be present in some of these other species as well. Because each novel find will be annotated throughout all bacterial genomes, even challenging genomes will become easier to analyze as our genome analysis campaign progresses.

A total of 76 *C. novyi* unknown IGRs were selected for detailed evaluation. 32 of these IGRs were found to correspond to known protein-coding regions, and two were found to encompass known ncRNAs (tmRNA and tRNA^Arg^). Nearly all novel conserved motifs uncovered (Additional file 1: Table S1b) among the remaining unknown IGRs are confined to the Firmicutes phylum, predominantly in the Bacilli and Clostridia classes. However, some motifs are also present in the other classes of Firmicutes (Negativicutes, Erysipelotrichia, and Thermolithobacteria). A number of motifs are very narrowly distributed, and are found only in *C. novyi* and *C. botulinum*, suggesting these classes might have emerged more recently in evolution. Most notably, an excellent riboswitch candidate was discovered and called the *thiS* motif RNA. Preliminary biochemical and genetic analyses of this motif are described in a later section.

*The* Thermovirga lienii *genome. T. lienii*, a Gram-negative bacterium in the phylum Synergistetes, was originally isolated from hot oil-well production water from the North Sea [47]. Despite the fact that the %GC content of its genome is somewhat elevated, the known ncRNAs present in this species strongly cluster in a region with only a small number of unknown IGRs (Additional file 5: Figure S3). Included in this region are a variety of known riboswitches and other ncRNAs. These properties, coupled with the fact that *T. lienii* is from a phylum that is evolutionarily distant from other organisms analyzed, prompted us to examine this genome.

A total of 78 unknown IGRs were selected for analysis (Additional file 1: Table S1c), of which 29 were found to correspond to ORFs for known proteins. Another IGR was found to correspond to a CRISPR [48, 49] repeat sequence. Surprisingly, 31 of the remaining IGRs do not possess any homologs and are only found in *T. lienii*. These individual IGR sequences were each designated “unnamed”. The remaining findings range in their phylogenetic distribution, and include candidates with representatives in species from other phyla. Although most of these motifs have been classified as mobile/repeat sequence candidates, we consider one to be a novel riboswitch candidate called SLH (Fig. 3e) as briefly discussed below.

The SLH (surface layer homology) motif has 80 unique examples and is found in the Synergistaceae family, in Firmicutes (Clostridia and Negativicutes), and in metagenomic DNA sequences. Approximately 60% of SLH motif representatives are found upstream of genes coding for proteins that carry S-Layer homology domains, whereas the rest are upstream of genes coding for proteins of unknown function. Surface layers (S-layers) are formed by identical protein subunits that assemble into a monomolecular crystalline array on the cell surface of most Archaea and many bacteria [50]. SLH domains are involved in the attachment of S-layer proteins to the cell wall. It seems possible that the ligand recognized by the SLH riboswitch candidate could be a signaling molecule involved in cell wall homeostasis, such as the second messenger cyclic-di-GMP [51].

*The* Arcobacter sp. L *genome.* The bacterium *Arcobacter* sp. L (hereafter referred to as *Arcobacter*) is a Gram-negative organism in the Epsilon-proteobacteria class (Proteobacteria phylum). It is closely related to *Arcobacter butzleri*, and both *A. butzleri* and *Arcobacter* were isolated from a microbial fuel cell in which acetate was the only carbon source [52]. When presented with an external electron acceptor, both species metabolized acetate much more efficiently by transferring electrons extracellularly to the electron acceptor, a process known as exoelectrogenesis. *A. butzleri* and *Arcobacter* are the first exoelectrogenic species to have been discovered in Epsilon-proteobacteria [52]. This genome possesses relatively few ncRNA types, but a large number of these are riboswitches (Additional file 6: Figure S4).

A total of 38 IGRs were selected for detailed analysis (Additional file 1: Table S1d), of which 23 were found to overlap known ORFs, and another was found to overlap a gene for a known ncRNA (SRP RNA, Rfam [53] ID: RF00169). Again, most of the remaining novel IGRs are rare and phylogenetically confined to the *Arcobacter* genus, and thus remain unnamed. The last four IGRs carry novel motifs that range in their phylogenetic distribution, with members in Alpha-proteobacteria and Firmicutes (see Additional file 3: Supplementary text for additional details).

*The* Baumannia cicadellinicola *genome.* The endosymbiont *B. cicadellinicola* is in the Gamma-proteobacteria class, lives within the cells of the glassy-winged sharpshooter insect (*Homalodisca coagulate*), and coexists with another endosymbiont bacterial species *Sulcia muellerilives* (Bacteroidetes phylum). All three species are believed to have coevolved a mutually beneficial relationship [54]. *B. cicadellinicola* has a relatively small genome (686 kb), and possesses a minimal complement of genes and known ncRNAs (Additional file 7: Figure S5). It has been demonstrated through comparative genomics analyses that a loss of structured ncRNAs, particularly *cis*-regulatory motifs, has occurred in a number of endosymbiotic bacteria from various phyla [55]. This previous study also revealed that ribosomal protein leaders tended to be one of the few *cis*-regulatory ncRNA types remaining in the endosymbiotic genomes, which appears to also be true for *B. cicadellinicola*.

Due to the diminished size of the *B. cicadellinicola* genome, only 16 IGRs were selected for analysis (Additional file 1: Table S1e). Six of these IGRs overlap ORFs for known proteins*.* Nearly all other findings are only rarely represented and are phylogenetically confined to the Gamma-proteobacteria class, and thus remain unnamed. However, we identified a single representative of a novel and widespread riboswitch candidate called *corA*, as briefly discussed below.

The *corA* motif (Fig. 3f) has 325 unique representatives, which are all found among Gamma-proteobacteria genomes and metagenomic DNA sequences. Within Gamma-proteobacteria, the *corA* motif is found within a number of other endosymbionts, and in a number of pathogenic bacterial species including *E. coli*, *Klebsiella pneumoniae*, *Salmonella enterica*, and *Yersinia pestis*. Nearly all *corA* motif representatives are located immediately upstream of the *corA* gene that encodes proteins implicated in Mg^2+^ and Co^2+^ transport. There are two Mg^2+^-sensing and one Co^2+^-sensing riboswitch classes known to date [8]. Thus, it seems reasonable to speculate that the *corA* riboswitch candidate might also respond to Mg^2+^, Co^2+^, or another divalent metal cation.

### Preliminary experimental analysis of the *thiS* riboswitch candidate

**Fig. 4:**
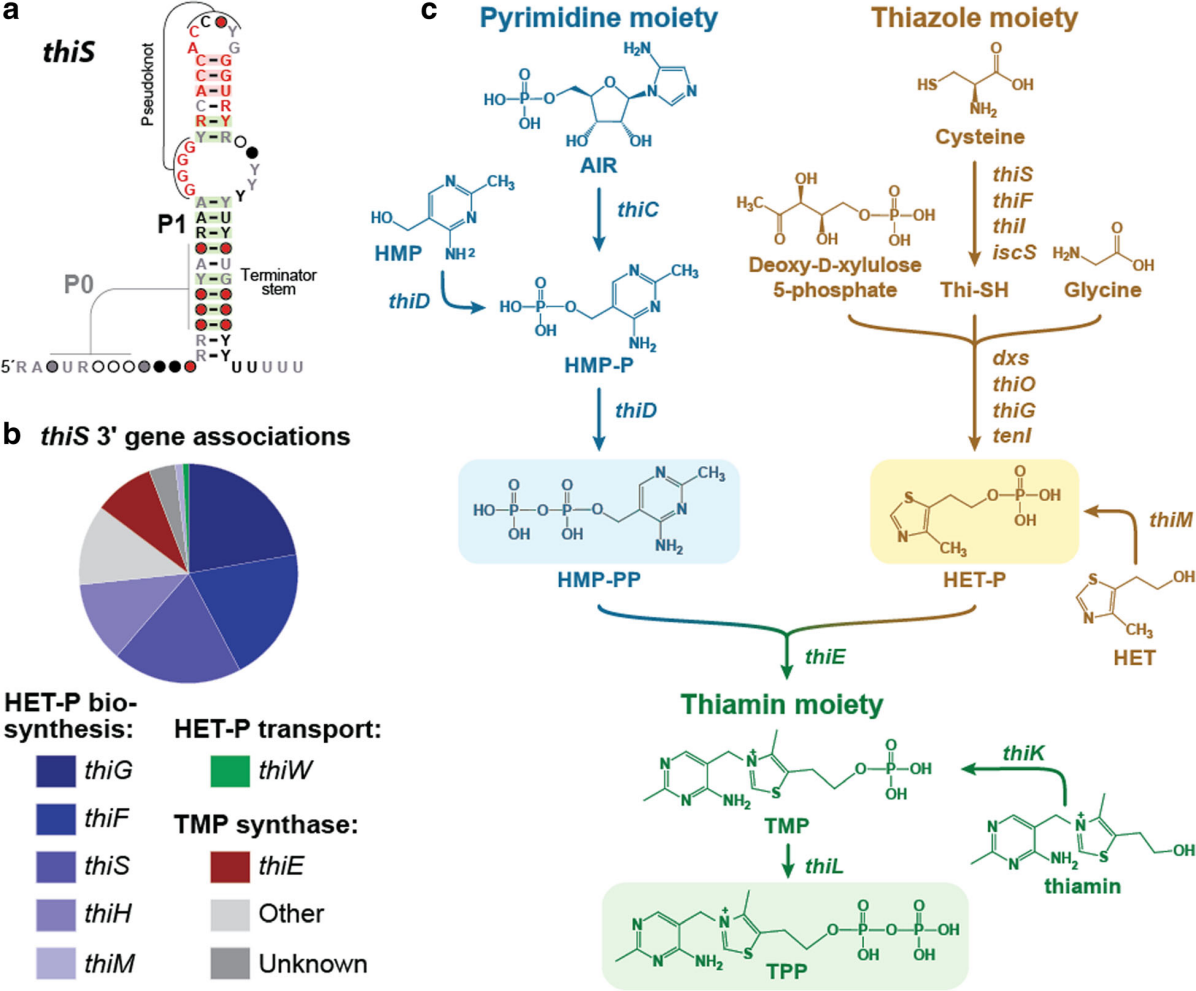
Structure and genetic context of the *thiS* motif. **a** Consensus sequence and secondary structure model for the *thiS* motif. Annotations are as described for Fig. 3. The P0 stem is predicted to exist if the lower portion of P1 fails to form. **b** (Top) Distribution of gene associations for the ~ 700 *thiS* motif representatives in bacteria. The chart incorporates the first five genes downstream of the *thiS* motif, and has a total of 1922 entries. These genes, which are typically in the *thiS* operon, are counted individually. (Bottom) Protein products of the genes abbreviated here, when known, catalyze the reaction steps for thiamin biosynthesis depicted in c. **c** The biosynthetic pathway of TPP in *Bacillus subtilis*. Acronyms starting from the top left are: aminoimidazole ribotide (AIR), hydroxymethyl-pyrimidine (HMP) hydroxymethyl-pyrimidine phosphate (HMP-P), hydroxymethyl-pyrimidine pyrophosphate (HMP-PP), hydroxyethyl-thiazole (HET), hydroxyethyl-thiazole phosphate (HET-P), thiamin monophosphate (TMP), and thiamin pyrophosphate (TPP). TMP (green shaded box) is formed by fusing the two compounds HMP-PP (blue shaded box) and HET-P (gold shaded box). Note that both HET-P and TPP can be synthesized through a salvage pathway starting with HET and thiamin, respectively. HMP-PP and HET-P were proposed as the top ligand candidates for the *thiS* riboswitch candidate. Metabolic scheme is based on that published previously [56]

The *thiS* motif RNA (Fig. 4a), originally identified by analyzing the *C. novyi* genome, has approximately 700 unique representatives that are found in Actinobacteria (7%), Firmicutes (27%), and metagenomic DNA datasets (66%). The motif is commonly found upstream of genes involved in the biosynthesis of TPP (Fig. 4b), which is an important coenzyme in all forms of life. Specifically, *thiS* motif RNAs are predominantly associated with genes involved in the synthesis of the thiamin precursor hydroxyethyl-thiazole phosphate (HET-P) [56] (Fig. 4c). In addition to its frequent association with *thiS* genes coding for the sulfur carrier protein ThiS, the *thiS* motif is also commonly found upstream of genes annotated as *thiW* (predicted HET-P transporter) and *thiE* (thiamin monophosphate (TMP) synthase), the enzyme that joins HET-P and hydroxymethyl-pyrimidine pyrophosphate (HMP-PP) to make TMP. Thus, we speculated that *thiS* motif RNAs might represent a novel riboswitch class that responds to a biosynthetic intermediate for TPP production.

Interestingly, ~ 20% of *thiS* motif RNAs reside immediately downstream of TPP riboswitches, whose members bind to the final product in the TPP biosynthetic pathway to control genes involved in synthesis of both the pyrimidine (HMP-PP) and thiazole (HET-P) precursors [57, 58]. Such putative tandem TPP and *thiS* motif RNA arrangements each carry their own expression platforms, which are RNA sequences and structures that interface with a riboswitch aptamer and the cell’s gene expression machinery (e.g. an RNA polymerase or a ribosome). These architectural features of tandem TPP riboswitch and *thiS* motif RNAs suggest that expression of the associated thiamin biosynthesis genes will be off when TPP is abundant, but that a second, distinct chemical input is evaluated when cells are starved for the TPP coenzyme. If true, these tandem arrangements would function as two-input Boolean logic gates, as has been observed for several other natural tandem riboswitch architectures that respond to two different ligands [15, 16].

**Fig. 5:**
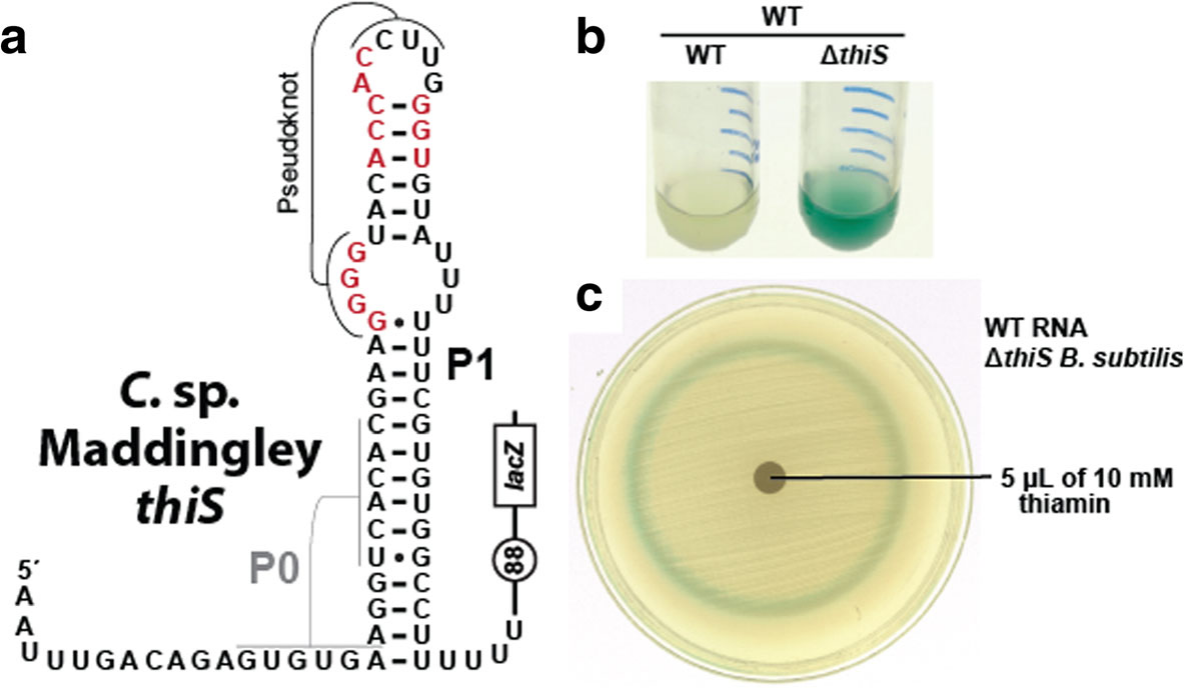
Reporter gene expression is regulated by the *thiS* riboswitch candidate. **a** Sequence and predicted secondary structure of the WT *thiS* RNA associated with the *thiS* gene of *C. maddingley*, which was fused to a β-galactosidase reporter gene (*lacZ*) and a *B. subtilis lysC* promoter to drive transcription. The *lysC* promoter was chosen for this purpose because it is known to strongly promote transcription without regulation [73]. Encircled 88 designates the number of additional nucleotides between the end of the terminator element and the *lacZ* reporter gene sequence. Red nucleotides are > 97% conserved in the *thiS* consensus model (Fig. 4a). **b** Reporter gene expression of WT *B. subtilis* cells and cells lacking the coding region for the ThiS protein (Δ*thiS*) grown in minimal (GMM) liquid media. **c** Agar diffusion assay of the Δ*thiS B. subtilis* strain with a WT riboswitch reporter construct. The filter disk was spotted with 10 mM thiamin on a minimal (GMM) agar medium plate with 100 μg mL^− 1^ X-Gal

To evaluate the potential regulatory function of *thiS* motif RNAs, we examined a representative that naturally lack an adjacent TPP riboswitch. Specifically, we examined a *thiS* representative from *Clostridium* sp. *Maddingley* by fusing its corresponding DNA sequence to a β-galactosidase (*lacZ*) reporter gene (Fig. 5a) and transforming this construct into *Bacillus subtilis* cells. Reporter strains were first grown in rich medium (containing sufficient thiamin), and then transferred by 1:20 dilution into minimal medium. No β-galactosidase activity is observed in minimal medium when the reporter construct expresses the wild-type (WT) *thiS* RNA element (Fig. 5b), suggesting that gene expression mediated by the *thiS* motif is naturally very low, even when thiamin might be biosynthesized in only small amounts by the host cells. In stark contrast, deletion of the *B. subtilis* gene coding for the ThiS protein (Δ*thiS*) yields robust reporter gene expression (blue color) when regulated by the WT *thiS* RNA element.

Presumably, cells grown in 1:20 rich to minimal liquid medium mixtures (volume to volume) have sufficient TPP to grow, but must begin to biosynthesize this coenzyme as it is further depleted from the medium. Under these conditions, Δ*thiS* cells trigger reporter gene activation unless the *thiS* motif RNA carries a critical mutation, suggesting that a change in the amount of the riboswitch ligand is promoted by the Δ*thiS* mutation. To further demonstrate that thiamin starvation triggers RNA-mediated activation of reporter gene expression we grew Δ*thiS B. subtilis* cells on a minimal medium agar plate supplemented with thiamin on a filter disk (Fig. 5c). This agar-diffusion assay demonstrates that these knock-out cells cannot grow without sufficient thiamin (outer clear ring). Moreover, Δ*thiS* cells that can still manage to grow but are deficient in TPP are experiencing conditions that activate gene expression by the *thiS* motif RNA (blue halo). Cells inside of the blue halo appear to grow well and do not experience the conditions needed to trigger gene expression changes mediated by the candidate riboswitch. These findings are consistent with our hypothesis that *thiS* motif RNAs function as *cis-*acting regulatory elements that respond to a molecular intermediate in the TPP biosynthesis pathway. Additional experiments are also consistent with a metabolite-binding riboswitch function for the *thiS* motif RNA class (Atilho RM, Mirihana Arachchilage G, Greenlee EB, Knecht KM, Breaker RR. A bacterial riboswitch class for the thiamin precursor HMP-PP employs a terminator-embedded aptamer. eLife. submitted).

## Concluding remarks

**Fig. 6:**
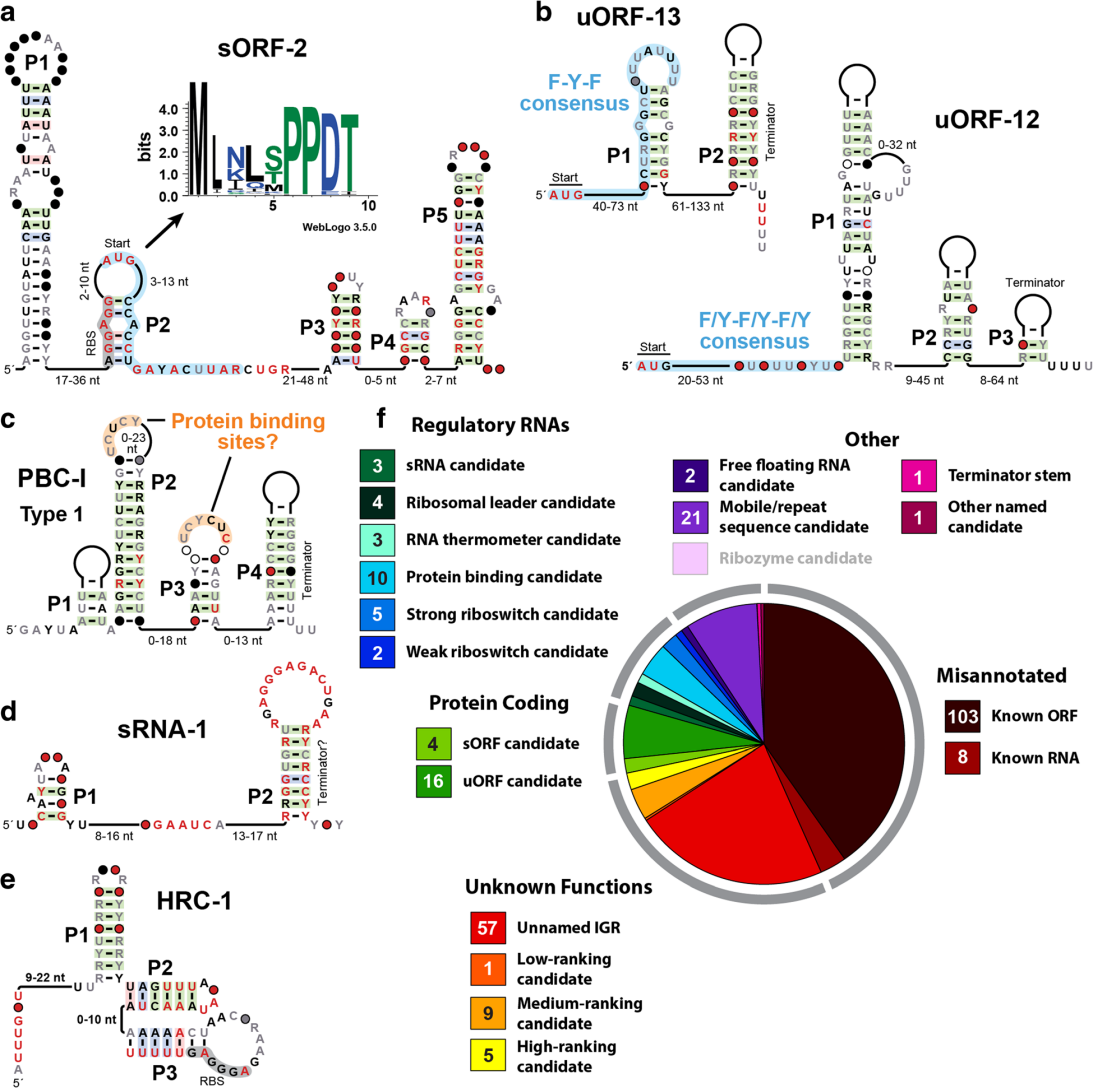
Representatives of various types of predicted structured nucleic acid motifs discovered among five bacterial genomes. **a-e** Sequence and predicted secondary structure models for representative ‘named’ motifs identified among the five bacterial genomes examined in this study. Extended blue shading in a and b designate possible short ORFs. For the translated WebLogo consensus sequence in a, amino acids in blue, green, and black are hydrophilic, neutral, and hydrophobic, respectively. The two candidate uORFs in b are associated with shikimate metabolism genes, and notable amino acids related to this pathway and encoded by the uORFs include phenylalanine [F] and tyrosine [Y]. The protein binding candidate in c is depicted with two pyrimidine-rich sequences highlighted that might function as protein binding sites. In addition to the type I consensus depicted, representatives conforming to a type II (only one hairpin-loop similar to P3) and a type III (terminator stem only) consensus also exist. RBS designates ribosome binding sites. Additional annotations are as described in the legend to Fig. 3. **f** Comprehensive summary of the fate of the unknown IGRs after analysis of the five bacterial genomes examined in this study. Annotations are as described in the legend to Fig. 2c

Our findings from the analysis of five bacterial genomes using the GC-IGR approach reveal that numerous candidate ncRNA motifs and many other genetic elements of unknown function can be efficiently discovered on a genome-wide scale. Although we have designed our bioinformatics pipeline to search for novel ncRNAs such as riboswitches, numerous other RNAs with diverse functions are also encountered. Some examples of motif classes with predicted functions different than that of riboswitches are presented in Fig. 6**,** Additional file 8: Figure S6, and Additional file 9: Figure S7. Frequently, we encounter RNAs that code for short peptides, which could either function as sORFs that code for tiny proteins with their own biological functions (Fig. 6a) or serve as regulatory uORFs (e.g. Fig. 6b). Similarly, we commonly observe RNA motifs with simple, repetitive structures that associate with putative nucleic acid binding proteins. We predict such motifs might be bound by protein factors that regulate gene expression (Fig. 6c). Some simple RNA structures are predicted to function as regulatory sRNAs (Fig. 6d). When no reasonable function can easily be proposed, we declare the class to be a high- (Fig. 6e), medium-, or low-ranking candidate based on characteristics discussed in more detail above.

All these additional RNA classes have the length and sequence characteristics that were sufficient for our computational pipeline to uncover them. However, it seems likely that many more RNAs that function as uORFs, sORFs, sRNAs and PBCs will have been missed because they do not always have the structural complexity that most other ncRNAs such as ribozymes and riboswitches require. Additional unusual motifs were uncovered by our bioinformatics pipeline, including very simple nucleic acid motifs (Additional file 10: Figure S8a,b) and larger motifs that appear to function as structured DNAs (Additional file 10: Figure S8c). In one instance, we identified a large structured motif (*sig70*) due to a fortuitous similarity to a relatively small motif from *Arcobacter sp. L* called HRC-4 (Additional file 11: Figure S9). However, these unusual discoveries are expected to be rare occurrences when compared to the frequency of finding other larger ncRNAs or RNAs that code for short peptides.

Frequently, we encounter motifs that have ambiguous clues regarding their biological and biochemical functions. For example, a motif we cautiously named PBC-10 (Additional file 9: Figure S7a,b) includes an intrinsic terminator stem and an association with genes in a manner that is consistent with a *cis*-regulatory function. The palindromic sequence near the origin of the P1 stem suggests that the PBC-10 motif might bind to a protein dimer and regulate the transcription of the downstream gene by preventing terminator stem formation. However, it remains possible that this palindromic region is incidental, and thus the RNA motif might employ a regulatory mechanism that does not involve protein binding (see additional comments in Additional file 3: Supplementary text). Given the diversity of structures and function revealed by this current search, it will be challenging to identify those candidates that most merit experimental analysis and to choose the most likely hypotheses to pursue.

Regardless of the future experimental challenges, we believe that the large-scale discovery of novel structured ncRNAs will be of considerable value. A comprehensive summary of our findings (Additional file 1: Table S1) is depicted in visual form in Fig. 6f. With this data, we can begin to estimate how many novel ncRNA candidates could be uncovered by using our GC-IGR analysis pipeline. Among the original collection of unknown IGRs from the five genomes, ~ 40% represent previously known genes coding for proteins or ncRNAs. About a quarter of the unknown IGRs have sequences or structural features that are rare, or that lack clues regarding possible functions, and so are not easily classified. Among the remaining IGRs are numerous RNAs that code for short peptides, and these types of RNAs are likely to be far more numerous because they are not readily uncovered by our search algorithm.

Finally, ~ 20% of the unknown IGRs in the region of special interest appear to be excellent structured ncRNA candidates with various functions, including novel riboswitches. These are of particular interest to us, as we have predicted that many thousands of novel riboswitch classes exist in nature [8]. Indeed, the identification of seven candidate riboswitch classes among the genomes analyzed for this study is consistent with our hypothesis that many more remain to be discovered and that the number of representatives for each newly-found riboswitch class should be quite small compared to most known classes. For each riboswitch candidate identified, clues regarding the possible ligand sensed can be derived from the identities of associated genes. Unfortunately, many bacterial genes code for proteins whose functions remain unclear, which will hamper experimental validation studies.

The GC-IGR discovery strategy, which is an expansion of past efforts [24, 25, 27] to exploit the relative abundance of G and C nucleotides in certain RNA genetic elements, provides a means to more comprehensively scour each genome for novel structured ncRNAs. Although we initially examined 2807 sequenced bacterial and archaeal genomes, not all are amenable to our analytical approach. However, the total number of sequenced genomes is certain to grow quickly, and therefore the GC-IGR search strategy can potentially be applied to thousands of genomes in the coming years. Moreover, this search approach is ideal for the discovery of rare ncRNAs, as the abundance of a particular class of ncRNA candidates is not the initial parameter used for identifying possible motifs for further study, as it is for some other bioinformatics search methods [4–6]. This distinction is important because the majority of common structured ncRNAs in bacteria almost certainly have already been discovered. Thus there is a need for a search approach adapted to efficiently and comprehensively discover the remaining rare structured ncRNAs and discern them from the large number of potential false positives.

Analysis of the first five genomes has served as a valuable exercise that reveals the types and numbers of novel nucleic acid motifs that can be expected to be uncovered by a continuation of this bioinformatics campaign. Nearly 70 novel genetic elements with proposed functions (Additional file 1: Table S1; Additional file 8: Figure S6f) have been predicted to be present in these genomes. Moreover, our initial efforts reveal some limitations to our search strategy, including a need for additional computational power, the time involved in carefully evaluating each motif to define a consensus sequence and secondary structure model, and a lack of information necessary to generate a reasonable hypothesis for the function of each motif.

Regardless, continued methodological improvements for genome and motif analysis should permit us to rapidly discover a large diversity of novel structured ncRNAs. These ncRNAs will provide insight regarding the varied roles and capabilities of RNA molecules, and some might potentially serve as targets for the development of new classes of antibacterial agents. Finally, comprehensive discovery of ncRNAs among the genomes amenable to this analytical approach will allow us to more accurately estimate the number of ncRNAs that exist in the bacterial and archaeal genomes that have yet to be fully sequenced.

## Methods

### Genome plotting, ranking, and IGR selection

Processing of IGR data from fully sequenced bacterial and archaeal genomes from RefSeq [29] was conducted as previously described [27] with some modifications as described below. RefSeq 63 provided the most recent data for known ncRNAs at the beginning of our project, therefore our initial list of genomes considered for analysis included ~ 2800 genomes available at that time. Genomes selected for in-depth analyses were chosen from those that provided reasonable separation of known ncRNA motifs from the majority IGRs (e.g. see Fig. 2a). As individual motifs were examined in greater detail, to establish the number of representatives and the consensus sequence and structural models, RefSeq 76 was adopted as the most recent available resource for DNA sequence data analysis. IGRs containing previously known ncRNAs were annotated based on information from Rfam [53] version 12, from RefSeq, and from a list of novel ncRNA motifs more recently identified by our laboratory [43].

The computer codes used to calculate IGR properties, rank genomes, plot IGRs, identify sectors of interest, generate selected IGR lists, and generate motif gene context frequency charts were written in MATLAB (The MathWorks, Inc., Natick, Massachusetts, United States). Candidate IGRs were selected by drawing a boundary directly on the plot to accommodate variability in IGR distributions for each genome (Fig. 1a). Characteristics regarding each IGR contained within the boundary were added to an Excel spreadsheet. This information included accession number and nucleotide position, IGR length, IGR %GC, 5′ and 3′ gene associations, and 5′ and 3′ gene orientations relative to the IGR. IGRs were ranked by their length in descending order, but all characteristics were considered when formulating hypotheses regarding the structures and functions of each candidate.

### Selected IGR analysis pipeline

Unknown IGRs present in the sector of interest were subjected to detailed analyses as schematically depicted in Fig. 1b-f and Additional file 2: Figure S1. The following paragraphs provide details regarding each of the bioinformatics analyses. Many of the steps described below have been automated using a script written in Python 3 (Python Software Foundation, https: //www.python.org/).

*Screen IGRs for overlapping proteins* (Fig. 1b and Additional file 2: Figure S1a,b). All selected IGR sequences were examined using BLASTX (nr/nt databases) [59] (https://blast.ncbi.nlm.nih.gov/Blast.cgi) to search for IGRs that overlap protein-coding regions. Any IGR that overlaps at least one named protein (known or predicted function) or two unnamed proteins (unknown function) with an alignment score above 50 is annotated as overlapping a protein and is removed from the analysis. IGRs partially overlapping protein-coding regions are also removed from analysis unless the flanking genes hint at the existence of a possible riboswitch, ribozyme, or other ncRNA. On removal of a protein coding region, if the remaining noncoding IGR portion has IGR length or %GC values that fall outside of the original sector of interest, it is removed from further consideration.

*Homology search and initial IGR categorization* (Fig. 1c and Additional file 2: Figure S1c). Remaining IGRs were analyzed using Infernal [60] version 1.1 on the most current RefSeq [29] version installed on the Yale HPC cluster (version 76 for runs described in this work), and a number of metagenomic environmental datasets described previously [61]. IGR homology search results in which the majority of sequences overlap protein-coding regions are cause for removal from the list of unknown IGRs to be extensively analyzed. The nr/nt databases used when running BLASTX in the previous step do not include protein-coding regions from all metagenomic datasets. Therefore the datasets used by our laboratory for Infernal runs include a number of metagenomic datasets and ORF sequences for other predicted proteins [22]. IGR homology search results that overlap known RNAs are annotated as such and added to the known RNA dataset for IGR plotting. If IGR results overlap short (< 100 nt) ORFs for “hypothetical proteins” they are retained for further analysis. Most often these IGRs contain attenuator or short ORFs. Other IGR homology results that do not overlap ORFs or known ncRNAs but possess other previously-known features are labeled accordingly and are not retained for further analysis.

*Manual alignment curation and motif direction determination* (Additional file 2: Figure S1d). For remaining IGR results, alignment (Stockholm) files are generated by use of cmalign, a component of Infernal (see user’s guide), and are manually inspected using the alignment editing tools RALEE [62] for false positive sequences which are then removed from the alignment file. The entire IGR sequence is used as input for the homology search, but conserved motifs typically do not span the entire IGR [63]. Thus, sequence homology generally decreases near one or both of the alignment boundaries of the IGR. Alignments are trimmed on either side of the putative motif until a conserved region is reached. Any sequences truncated more than 50% after trimming of the alignment are removed. Next, motif direction is determined as depicted in greater detail in Additional file 2: Figure S1d. All IGRs submitted for analysis are on the ‘forward’ strand by default.

*Open reading frame search* (Additional file 2: Figure S1e). To determine if an upstream open reading frame (uORF) [34, 35] or a short open reading frame (sORF) [36, 37] is present in the conserved motif, all alignments were run through RNAcode with default settings [64]. Any alignments that contained a predicted ORF were manually examined for an RBS sequence (five to eight purines approximately eight nucleotides upstream of the start codon) and to see if additional ncRNA structure is possible other than an intrinsic terminator stem. If an RBS is found and there appears to be no possibility of additional secondary structure except a terminator stem, the motif is labeled as a uORF if gene context indicates possible *cis*-regulation by the ORF, and sORF if gene context appears independent of transcription of the ORF. Motifs that contained an ORF but also possessed the potential for forming RNA structures were retained for further analysis. In some cases (e.g. attenuator candidates), the sORF or uORF was bioinformatically translated and aligned using TranslatorX [65] and a consensus of the polypeptide sequence was created using Weblogo 3 [66].

*Structure prediction and top predicted structure(s) selection* (Fig. 1d and Additional file 2: Figure S1f). To derive structure predictions, candidate motifs remaining at this stage were analyzed by the CMfinder [67] algorithm on computer nodes maintained by the Yale Center for Research Computing. Identical sequence representatives, commonly resulting from the fact that the genome sequences of closely related strains are present in the DNA sequence databases, are removed such that only representatives with unique sequences are compared. CMfinder results typically include 5 to 10 potential structure predictions for a candidate motif. Often multiple predictions include some of the same substructures, but each include slightly different features. In these cases, one of the predictions was chosen as most reasonable, and the related models were removed. In some cases, stems contain multiple A-C mismatches, which often indicates that the alignment is in wrong orientation because these A-C mismatches become G-U base-pairs when transcribed from the opposite strand. When strong evidence exists for a particular orientation, only the correct orientation and corresponding structure prediction was pursued.

Next, alignments were analyzed by R-scape (RNA Significant Covariation Above Phylogenetic Expectation) [68] with default settings (R-scape User’s Guide) to provide an independent structure prediction and assist with choosing the best structure from the different predictions provided by CMfinder. If R-scape yields a very poor structure (only a few base pairs forming stems 1–3 base-pairs long with no covariation) or no structure, CMfinder results for that motif were reexamined. If structure predictions by CMfinder were equally poor, implying little or no structure, motifs were labeled as low-ranking candidates (LRC). If CMfinder results contain more significant structure, even if it contains little or no covariation, the motif was still retained for further analysis.

For remaining motifs, CMfinder structure predictions were manually examined for structure quality as judged by the presence of substantial covariation, conserved nucleotides in unpaired regions, well-structured G-C-rich stems (rather than stems made only of runs of A and U nucleotides), multi-stem junctions, potential pseudoknots [69], and other common RNA motifs such as GNRA or UNCG tetraloops and kink-turns [70, 71]. They were also compared to R-scape results for insight on significant sections of the structures. After manual analysis, the top one or two CMfinder structure predictions were selected and their alignment files manually edited. False positive sequences and those truncated more than 50% were removed, and an R2R consensus model [72] of the alignment was generated (Fig. 1e and Additional file 2: Figure S1f).

Some of these remaining motifs have few representatives and modest evidence for structure formation, and were classified as medium-ranking candidates (MRC) (see Additional file 2: Figure S1f for details). If lack of covariation does not seem to be derived from a low number of similar sequences, the motif is examined for open reading frames by running a few sequences through a translation tool (fr33.net/translator.php). If a conserved open reading frame is present, the motif is labeled as a uORF or sORF.

*Terminator stem analysis* (Additional file 2: Figure S1 g). Motifs containing terminator stems were subjected to an additional stage of the analysis pipeline. First, the motif alignment was trimmed to remove the right shoulder of the terminator stem, and reexamined by using Infernal. Motifs containing structural features in addition to the terminator stem (and all remaining motifs that did not contain a terminator stem) proceeded to the next step.

*Final bioinformatics motif analysis and function prediction* (Fig. 1f and Additional file 2: Figure S1 h). The next stage of analysis involved iterations of the homology search and structure prediction steps to further refine the consensus sequence and structural model, and to find additional homologous sequences. Further manual curation of the alignment files were performed to search for additional covariation, conserved nucleotides and/or short repeating sequences, common RNA structural units, and alternative structures. In some cases, motif boundaries were determined via discovery of the transcriptional start site, the RBS, or the start codon. If the termini of the motif remained undetermined, alignments were expanded in one or both directions to search for additional conserved sequences and structures. Iterations of the analytical stages between Infernal and CMfinder applications were performed two to four times, but sometimes this process was repeated six or more times (e.g., for the *thiS* motif), depending on the number of representatives, the complexity of structure, and the predicted function.

During or after motif refinement, the function of a motif was predicted based on genetic context and/or motif features, such as conserved repeat sequences. See Additional file 2: Figure S1 h for more details. If a motif function was evident but not listed in the IGR pipeline flowchart, the motif was named according to the predicted function. If the function is unknown, the motif is labeled as a high- (HRC), medium- (MRC), or low-ranking candidate (LRC), or sometimes is named after a common gene association if evident. All findings within the analyzed IGRs, including unannotated known ncRNAs and sORFs, are added to the dataset of known motifs for the plotting algorithm using another script written in MATLAB.

### Genetic assays for *thiS* riboswitch candidate

The *B. subtilis* riboswitch-reporter construct containing the *lysC* promoter from *B. subtilis* and *thiS* motif from *C. maddingley* (ALXI01000155.1) was synthesized by Integrated DNA Technologies and inserted into the vector pDG1661 via restriction digest and transformed into *B. subtilis* strain 168 1A1 or *thiS* knockout (∆*thiS) B. subtilis* strain. *B. subtilis* riboswitch-reporter strains were grown overnight at 37 °C in the rich medium Lysogeny Broth (LB). For liquid-culture reporter assays, the strains were then diluted 1/20 into glucose minimal medium (GMM). The residual thiamin from LB is sufficient for growth in GMM over the timescale of the assay (overnight at 37 °C). GMM liquid media was supplemented with X-gal (100 μg mL^− 1^) to allow detection of reporter gene expression.

For agar-diffusion assays, cells grown in LB were spun down and washed with GMM to remove residual thiamin, and then were spread on GMM agar plates supplemented with X-gal as noted above. Autoclaved paper discs prepared from pure cellulose chromatography paper from Fisher Scientific were soaked with 5 μL of 10 mM thiamin and transferred to agar plates. Plates were incubated overnight at 37 °C.

## Additional files

Additional file 1: **Table S1.** Additional details regarding each motif and the summary of findings from all five genomes are presented in this table. (XLSX 44 kb)

Additional file 2: **Figure S1.** Schematic flowchart for the GC-IGR analytical pipeline. (PDF 823 kb)

Additional file 3: Supplementary text. This file includes descriptions of additional named motifs and select high ranking candidates. (PDF 143 kb)

Additional file 4: **Figure S2.** Plots of the IGRs from the *C. novyi* genome sorted based on IGR length and GC content. (PDF 131 kb)

Additional file 5: **Figure S3.** Plots of the IGRs from the *T. lienii* genome sorted based on IGR length and GC content. (PDF 120 kb)

Additional file 6: **Figure S4.** Plots of the IGRs from the *A. sp. L* genome sorted based on IGR length and GC content. (PDF 95 kb)

Additional file 7: **Figure S5.** Plots of the IGRs from the *B. cicadellinicola* genome sorted based on IGR length and GC content. (PDF 66 kb)

Additional file 8: **Figure S6.** Consensus sequence and secondary structure models for additional structured ncRNA motif candidates discovered in this study. (PDF 157 kb)

Additional file 9: **Figure S7.** Consensus sequences and secondary structure models for additional ncRNA motif candidates or candidates with other functions discovered in this study. (PDF 385 kb)

Additional file 10: **Figure S8.** Consensus sequence and secondary structure models for small ncRNA motif candidates and a candidate ssDNA motif. (PDF 69 kb)

Additional file 11: **Figure S9.** Fortuitous discovery of a large structured motif. (PDF 136 kb)

## Abbreviations

A: Adenine
*Arcobacter*: *Arcobacter sp. L*
*B. cicadellinicola*: *Baumannia cicadellinicola*
C: Cytidine
*C. novyi*: *Clostridium novyi*
G: Guanosine
GMM: Glucose Minimal Medium
HET-P: Hydroxyethyl-Thiazole Phosphate
HET-P: hydroxyethyl-thiazole phosphate
HIMB5: alpha proteobacterium HIMB5
HMP-PP: Hydroxymethyl-Pyrimidine Pyrophosphate
HRC: High Ranking Candidate
IGR: Intergenic Region
LB: Lysogeny Broth
LRC: Low Ranking Candidate
MRC: Medium Ranking Candidate
ncRNA: Noncoding RNA
ORF: Open Reading Frame
*P. ubique*: *Pelagibacter ubique*
RBS: Ribosome Binding Site
SLH: Surface Layer Homology
sORF: Short Open Reading Frame
SRP: Signal Recognition Particle
T: Thymidine
*T. lienii*: *Thermovirga lienii*
tmRNA: transfer-messenger RNA
TPP: Thiamin Pyrophosphate
U: Uridine
uORF: Upstream Open Reading Frame
WT: Wild Type
