# Supplementary material for: Genome-wide discovery of structured noncoding RNAs in bacteria
Source: BMC Microbiol. 2019 Mar 22;19:66. doi: 10.1186/s12866-019-1433-7 (PMC6429828; doi:10.1186/s12866-019-1433-7)
Supplement: Supplementary file 2 — Figure S1. Schematic flowchart for the GC-IGR analytical pipeline. (PDF 823 kb) [file 12866_2019_1433_MOESM2_ESM.pdf]

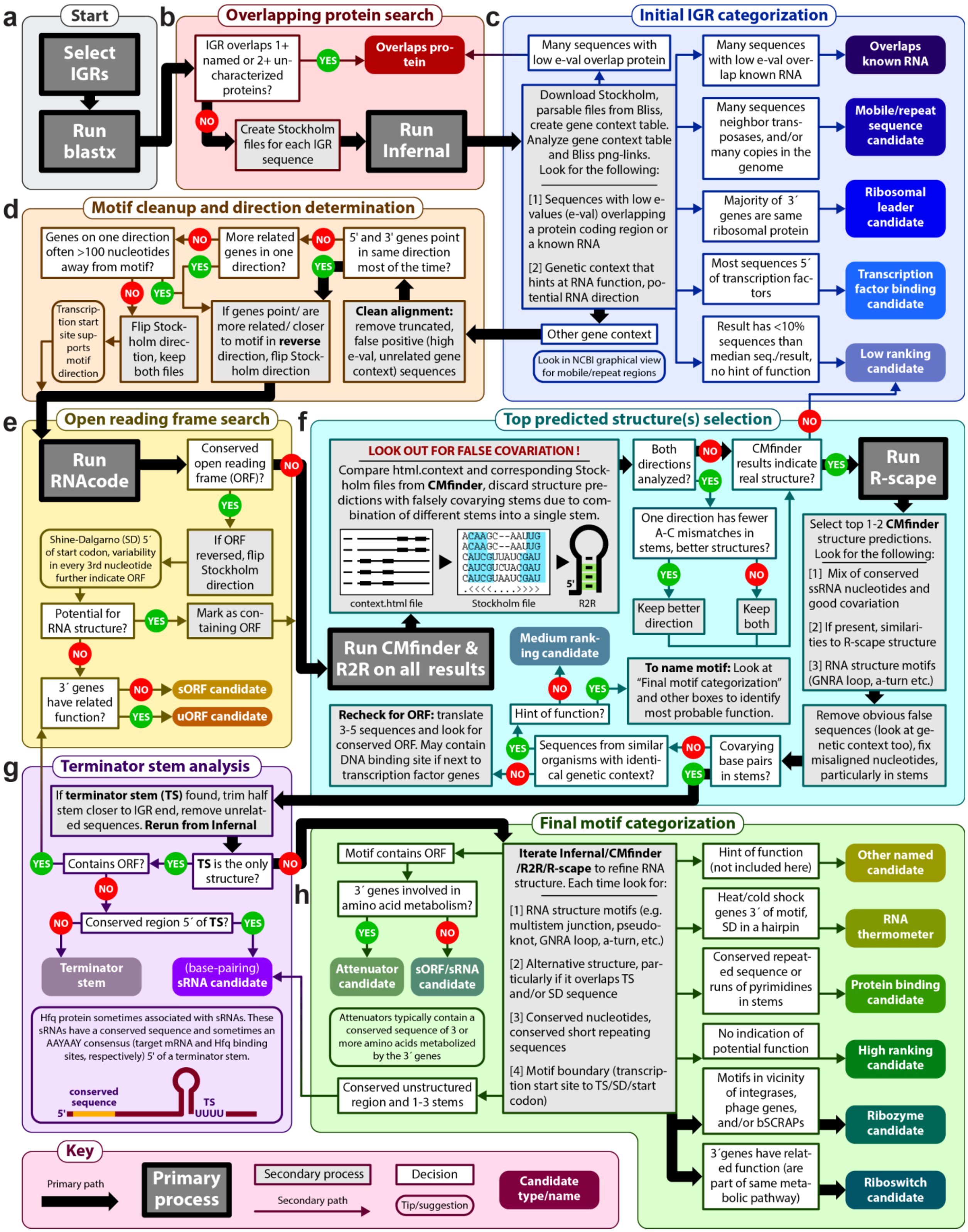

Figure S1 | Detailed flowchart of the GC-IGR analytical pipeline. Detains on stages a through h are provided in the Methods section.
