# Supplementary material for: Genome-wide discovery of structured noncoding RNAs in bacteria
Source: BMC Microbiol. 2019 Mar 22;19:66. doi: 10.1186/s12866-019-1433-7 (PMC6429828; doi:10.1186/s12866-019-1433-7)
Supplement: Supplementary file 3 — Supplementary text. This file includes descriptions of additional named motifs and select high ranking candidates. (PDF 143 kb) [file 12866_2019_1433_MOESM3_ESM.pdf]

BMC Microbiology

Supplementary Text

Genome-wide discovery of structured noncoding RNAs in bacteria

Shira Stav[1], Ruben M. Atilho[2], Gayan Mirihana Arachchilage[3], Giahoa Nguyen[1], Gadareth Higgs[1], and Ronald R. Breaker[1,2,3*]

[1]Department of Molecular, Cellular and Developmental Biology, [2]Department of Molecular Biophysics and Biochemistry, [3]Howard Hughes Medical Institute, Yale University, New Haven, Connecticut 06520, United States.

*e-mail: ronald.breaker@yale.edu

twitter: @RonBreaker

Descriptions of Additional Named Motifs and Select High Ranking Candidates

The sORF-2 motif. The sORF-2 motif (**Fig. 6a**) identified in HIMB5 has 802 unique examples. Members of this RNA class have features that are similar to transcription attenuator systems, such as a short ORF (with a highly-conserved PPDT polypeptide sequence) adjacent to RNA hairpin structures (**Fig. 6a**). However, unlike transcription attenuator elements, this motif does not appear to have any specific gene association, as both flanking genes are diverse (often of unknown function). Moreover, the coding regions of these flanking genes often are oriented toward the RNA element, and thus are not in a position to be regulated as a single RNA transcript. The motif might represent a *trans*-acting element consisting of both an sRNA and an sORF, which could explain the presence of a short coding region typical of an sORF and more complex RNA structure typical of sRNAs.

Aromatic amino acid attenuators. Two predicted RNA motifs discovered in *C. novyi* are found predominantly upstream of genes involved in biosynthesis of the aromatic amino acids tyrosine and phenylalanine via the shikimate pathway and tyrosine/phenylalanine biosynthetic pathways beginning with chorismate mutase [1], and were named uORF-12 and uORF-13 (**Fig. 6b**).

The uORF-12 RNA has 1258 unique representatives, of which 90% are in Firmicutes and the remaining in metagenomic environmental sequences. Unlike genes associated with uORF-13 RNA, the majority of genes downstream of the uORF-12 RNA encode amino acid transporters, but there is some overlap in genetic context with the uORF-13 RNA, including genes for the shikimate and tyrosine/phenylalanine biosynthesis pathways. The structure of uORF-12 is more complex than that of uORF-13, and the F-Y-rich peptide is encoded in a short region immediately preceding stem P1. In addition to the different structures and genetic contexts of the two shikimate-associated RNAs, their genetic contexts vary greatly within the Firmicutes phylum. Only a number of *Clostridium* species (*C. aceticum, C. botulinum, C. cadaveris, C. difficile, C. novyi, C. perfringens, C. sartagoforme*) and three other species under the Clostridiales order (*L. bacterium, T. glycolicus, T. othiniensis*) contain both such motifs.

The uORF-13 RNA (**Fig. 6b**) has 336 unique examples, of which 43% are found in Firmicutes and the remaining in metagenomic environmental sequences. Approximately half of the sequences are upstream of *aroF* that encodes the tyrosine-sensitive 3-deoxy-D-arabino-heptulosonate 7-phosphate (DAHP) synthase, which catalyzes the first step in the shikimate pathway. The next

most commonly associated gene is *tyrA*, which is in the same operon as *aroF* and also involved in tyrosine biosynthesis. Other relevant genes found downstream of the RNA motif code for amino acid transporters, shikimate-3-phosphate synthase, shikimate kinase, and shikimate 5-dehydrogenase [2]. The consensus model of this motif includes a short ORF upstream of a terminator stem. This short polypeptide contains a conserved F-Y-F consensus (the aromatic amino acids tyrosine and phenylalanine) within the loop of P1. Because this short peptide is encoded upstream of genes involved in the biosynthesis of tyrosine, we believe this short polypeptide and terminator stem form a transcription attenuator, similar to the attenuator previously found upstream of the *trp* attenuator that controls the *trp* operon (tryptophan biosynthesis) [3].

The uORF-12 and uORF-13 RNAs are also structurally and phylogenetically distinct from and *aroR*-associated attenuator found in Actinobacteria. This RNA, recently discovered in *Corynebacterium glutamicum* and a few other Actinobacteria through transcriptomics [4], also contains a conserved F-Y-F amino acid consensus, but the rest of the short peptide and secondary RNA structure of the *aroR* element are distinct from those of the shikimate-associated motifs, and the RNAs are confined to different bacterial phyla.

The PBC-I motif. The PBC-I candidate (**Fig. 6c**) has 1418 unique representatives that can be separated into three types (1, 2 and 3) with a distribution of 686, 704 and 28 examples, respectively. Each type is a truncated version of the previous type. Type 1 consists of four stems, wherein P4 appears to be an intrinsic terminator stem. Two of the stems (P2 and P3) each contain a run of pyrimidines (alternating CUCUCU nucleotides, orange shading), that might serve as protein binding sites, as has been observed for polypyrimidine tract binding proteins [5]. Type 2 only consists of two hairpin structures analogous to the P3 and P4 hairpins from type 1, with a single run of pyrimidines. Type 3 consists of only the terminator stem found in the first two types. These representatives are almost always downstream of or between two ammonium transporter genes annotated as *TIGR03644*, and thus might function as protein-binding domains that regulate expression in response to cellular nitrogen levels.

The sRNA-1 motif. The sRNA-1 candidate (**Fig. 6d**) has 1038 unique representatives. In almost all cases, both flanking genes point towards the IGR and have various functions, so this motif is likely not a *cis*-acting RNA. This motif contains a highly conserved unstructured sequence (GAAUCA) found between two conserved hairpin structures. These architectural features are similar to those of known sRNAs [6]. Notably, this RNA also carries a highly conserved sequence

(GAGGGAG) in the loop of P2 that is the exact complement of the conserved sequence in the PBC-3 RNA (Additional file 8: Figure S6c). The flanking nucleotides to both of these sequences are not as well conserved, but also nearly perfectly base-pair. The fact that both the PBC-3 RNA and this sRNA candidate have nearly the same number of unique sequences and are present in the same genomes is further evidence that these two RNA candidates might function together.

The HRC-1 motif. HRC-1 (High Ranking Candidate 1) (**Fig. 6e**) was first identified in HIMB5, and there are a surprising 956 unique examples. It seems most likely that the orientation depicted is biologically relevant because it includes the ribosome binding site of the downstream gene. In this orientation, the commonly associated ORF encodes bacteriorhodopsin-like protein (biphenyl 2,3-dioxygenase), a light-dependent proton pump involved in energy production. However, it is known that expression of the ORF does not vary under changing light conditions in *P. ubique* [7], and it is not believed to function in response to light in the SAR11 clade [8].

The HRC-2 motif. This candidate (Additional file 8: Figure S6a) has 639 unique examples and is routinely located upstream of the *amtB* gene, which encodes a membrane ammonium/ammonia channel. There is debate on whether this transporter channels the ammonium ion or neutral ammonia, but there is evidence for the latter [9]. The structure is rather unusual for a single regulatory motif, and consists of a conserved GUUGAU sequence followed by a possible terminator stem (P1), followed by another stem (P2) and additional conserved nucleotides. The possible terminator stem, which appears to be unusually small, might belong to the upstream gene (unknown function) and thus leave little structure left for an adjacent regulatory element. Alternatively, it is possible that the GUUU sequence at the 5´end base-pairs with the AAAC sequence closely upstream of the RBS sequence, in which case the P1 hairpin will be part of a larger RNA motif that might function as a *cis*-regulatory element.

The HRC-3 motif. The HRC-3 motif (Additional file 8: Figure S6b) identified in the *C. novyi* genome is represented by 77 unique examples. All but two sequences are in the Clostridiaceae family, of which nearly all are from *Clostridium* species. Nearly all sequences are found upstream of the *murD* ORF that encodes UDP-*N*-acetylmuramoyl-L-alanyl-D-glutamate ligase and is involved in peptidoglycan biosynthesis [10]. Three sequences are upstream of the uracil-DNA glycosylase gene (found only in thermophiles) followed by *murD*. The HRC-3 motif contains a reverse terminator stem (P1), but both flanking genes point in the forward direction, so a putative terminator stem on the reverse strand would not belong to either of the flanking genes. Another

possibility is that the HRC-3 motif represents a *cis*-regulatory antisense RNA regulating the *murD* gene through transcription termination, in which case the terminator stem is transcribed on the reverse strand [11].

The PBC-3 motif. This candidate (Additional file 8: Figure S6c) has 1111 unique examples and is found upstream of the gene that encodes CmcC, a cytochrome c assembly protein which is part of the membrane-associated cytochrome c maturation system found in most Gram-negative bacteria [12]. The PBC-3 motif RNA consists of a single, large, highly covarying stem with a highly conserved pyrimidine rich sequence CUCCCUC, which may also be a binding site for a type of polypyrimidine tract binding protein [5], or perhaps this sequence serves as a binding site for an sRNA. Regardless, this motif appears to be *cis*-regulatory, as it is almost always upstream of the same gene.

The SAR11-groES *motif*. This candidate (Additional file 8: Figure S6d) has 722 unique examples, all upstream of the gene coding for GroES. The SAR11-groES RNA has a structure vaguely similar to RNA thermometers [13], because both the RBS and start codon of the GroES ORF are occluded by a base-paired stem (P2). For additional information on motifs associated with *groES* genes, see the description for the Firmi-*groES* motif (Additional file 8: Figure S6i).

The ffRNA-1 motif. The ffRNA-1 (free-floating RNA, class 1) motif (Additional file 8: Figure S6e) has 4460 unique sequences, in many cases appearing multiple times in the same species. Approximately 75% of all representatives are in Proteobacteria; of these, 50% are in Betaproteobacteria, 44% in Gammaproteobacteria, 4% in Deltaproteobacteria, and 1.3% in Epsilonproteobacteria. Remaining sequences are found in Actinobacteria (only two species: *Bifidobacterium* and *Streptomyces pluripotens*), Bacteroidetes, Firmicutes (only in one species: *Megamonas rupellensis*), and in environmental DNA sequences. There is no common gene association for the ffRNA-1 motif, as genetic context varies greatly among various representatives. We have named this RNA (and another RNA called ffRNA-2) 'free-floating' because its role does not appear to be related to regulation of the neighboring genes, and thus it might have a biological function that requires it to diffuse away from its site of transcription. For this specific RNA, it seems most likely that it is part of a selfish genetic element.

The uORF-11 motif. There are only eight unique examples of the uORF-11 RNA candidate (Additional file 8: Figure S6f). In all cases, this motif is found between the genes coding for CoxG (carbon monoxide dehydrogenase subunit G) and CoxS (carbon monoxide dehydrogenase subunit

S). Due to the limited examples of this motif, it is difficult to be confident in the proposed secondary structure. There is a potential for a short ORF, but again the lack of examples precludes the use of bioinformatics to gather evidence for this possible function. However, the proximity of this putative RNA motif to the RBS of the downstream ORF suggests a *cis*-regulatory function.

The uORF-I motif. (Additional file 8: Figure S6g). This motif has 1064 unique examples, all upstream of the same ammonium transporter gene associated with the PBC-I motif. This motif contains a combination of RNA secondary structure and a short ORF (see shading). Because this motif is commonly located upstream of an ammonium transporter gene, it is likely a *cis*-regulatory element. The amino acid immediately after the initial methionine (black M) is a highly conserved glutamine (blue Q), and glutamine requires ammonium ions for its synthesis. Ammonium homeostasis might be particularly challenging for organisms in the SAR11 clade considering the nutrient-poor environments in which these bacteria reside [14]. This uORF motif might act as a glutamine-sensing control mechanism to increase uptake of ammonium by expressing more of the ammonium transport proteins.

The Clost-cspC *motif*. *Clost-cspC* (Additional file 8: Figure S6h) appears to be an exceptionally large RNA but unfortunately only has 11 unique examples, all found within the closely related species *C. botulinum*, *C. haemolyticum*, and *C. novyi*, and among environmental DNA sequences. In all cases, it is found upstream of *cspS*, which encodes a cold shock protein. Because the *Clost-cspC* motif has only a few very similar sequences, it is difficult to validate the proposed secondary structure model due to limited evidence for covariation. However, it appears to contain a stem (P9) that occludes the RBS and start codon of *cspC*, which is a feature of known RNA thermometers [13, 15]. The *cspC* gene is homologous to *cspA*, and the existence of a distinct *cspA*-associated RNA thermometer is already known [16]. However, the *cspA* thermometer has a different structure than *Clost-cspC* and is much more widely distributed. It is now known that *cspC* plays a role in reacting to other stressors such as NaCl, pH and ethanol [17], and thus it is possible *Clost-cspC* responds to a stressor other than temperature.

The Firmi-groES *motif*. Firmi-*groES* (Additional file 8: Figure S6i) is represented by 98 unique sequences and is found predominantly in the Clostridia class (83%), but also in Bacilli (4%) and the rest in environmental DNA sequences (13%). This motif is found upstream of the gene for GroES, a membrane-bound heat-shock chaperonin protein whose expression is sometimes controlled by an RNA thermometer [13] and is also associated with another conserved RNA motif

called Pseudomon-*groES* RNA and published previously [18]. The proposed secondary structure of Firmi-*groES* includes two extended base-paired structures wherein hairpin P2 includes nucleotides of the RBS and start codon for the downstream ORF. Thus, the secondary structure as presented should suppress expression of the *groES* gene unless a biochemical or physicochemical signal disrupts the formation of P2.

The PBC-10 motif. The PBC-10 motif (Additional file 9: Figure S7a) was initially identified in *Thermovirga lienii*. A total of 53 unique examples have been uncovered mostly in species of Synergistaceae and metagenomic DNA sequences, but one example has been found in Actinobacteria. Although the secondary structure of this motif contains very few conserved nucleotides, it has the potential to form alternative structures including a terminator stem and a possible pseudoknot. The PBC-10 motif is always oriented in the same direction as the immediately downstream gene, suggesting it has a *cis*-regulatory function. Notably, the genes that are mostly associated with the motif encode amino acid transporters or are involved in amino acid metabolism (Additional file 9: Figure S7b). The two most commonly associated genes, acetolactate synthase (*ilvH*) and phenylacetate-CoA ligase, are involved in branched-chain amino acid biosynthesis and phenylacetate catabolism, respectively. Oftentimes, the PBC-10 motif is found more than once in the same genome, but upstream of different genes. As discussed in the main text, this motif might function to bind a protein regulatory factor.

The sORF-1 motif. The first example of a candidate short open reading frame [19] (sORF-1 motif, Additional file 9: Figure S7c) we identified in our search has 1,080 unique examples. This sORF is preceded by a terminator stem that likely belongs to the gene located immediately upstream. The ORF (blue shading) is preceded by a modestly-conserved RBS sequence (gray shading). Notably, every third nucleotide is designated with an asterisk to highlight the fact that they are typically less conserved than the rest of the nucleotides, which is a hallmark of an ORF sequence. The predicted protein derived from this ORF is rich in both serine and lysine. The genes associated with this motif are diverse, and only occasionally positioned in the same orientation as the sORF. Therefore, it appears that the act of translation is not critical for gene control, as might be expected for a classic translational attenuation system. Rather, it appears that the short peptide might have a function that merits the conservation of this sORF.

The sORF-3 motif. sORF-3 (Additional file 9: Figure S7d) has 398 unique examples. This sORF candidate is followed by a terminator stem, which is the only notable secondary structure

feature, and which likely terminates the sORF transcript. Thus, only the lysine-rich peptide product is shown. In approximately 80% of the examples, the genes located immediately upstream are unknown due to truncation of the DNA sequence reads. The gene most often located downstream (approximately 50% of the examples) is COG1329, which encodes an RNA polymerase-interacting regulator of the CarD/CdnL/TRCF family. It is transcribed on the reverse strand relative to the sORF, and therefore this motif is unlikely to be a *cis*-regulatory RNA.

The uORF-5 motif. A total of 118 unique representatives of another upstream open reading frame, called uORF-5, were identified. Its expected peptide product (Additional file 9: Figure S7e) is enriched in the positively charged amino acids arginine and lysine. The uORF is most often located downstream of *mnmA*, which encodes tRNA(5-methylaminomethyl-2-thiouridylate)-methyltransferase, and upstream of the gene coding for cystathionine beta-lyase/cystathionine gamma-synthase. Due to the direction of transcription relative to the neighboring genes, this motif appears to be an attenuation system involving a *cis*-regulatory element that exploits translation of a short peptide to regulate transcription of the main ORF located immediately downstream.

The RPL-1 motif. The first example of ribosomal protein leader candidate (RPL-1 Additional file 9: Figure S7f) uncovered in our search was initially identified in the *C. novyi* genome. The RPL-1 motif has 4546 unique examples found in Firmicutes (all classes), Proteobacteria (Alpha-, Beta-, Gamma-, and Delta-), Haloplasmatales, Tenericutes, Thermotogae, and environmental DNA sequences. Several secondary structure models for ribosomal protein regulatory RNAs in *E. coli* have been reported [20]. However, the consensus sequence and the secondary structure model of RPL-1 is distinct from these previously reported ribosomal protein regulatory RNAs. Nearly all RPL-1 sequences are found upstream of the *rpsJ* gene that codes for 30S ribosomal protein S10. Thus, the RPL-1 motif might act to regulate ribosomal protein S10 biosynthesis.

The ffRNA-2 motif. The ffRNA-2 motif (Additional file 9: Figure S7g) is the second example of a 'free-floating' RNA class uncovered in our search. There are 621 unique examples of ffRNA-2 motif found in Proteobacteria (Alpha-, Gamma-, Delta-, and Epsilon-), Actinobacteria, Bacteroidetes and Firmicutes. Similar to ffRNA-1 motif, the genetic context of the ffRNA-2 motif varies greatly. This suggests the RNA is not a *cis*-regulatory element, but that it might be produced as a functional RNA that freely diffuses away from its site of transcription. Another possibility is that ffRNA-2 might be a part of a selfish genetic element. The secondary structure model of

ffRNA-2 exhibits some similarities to ffRNA-1, as both motifs have long P1 stems with conserved internal loops, although a P2 stem is absent in the ffRNA-2 structural model.

The RAT-1 motif. A ribonucleic anti-terminator candidate (RAT-1 motif, Additional file 9: Figure S7h**)** that has 766 unique examples was discovered in the *C. novyi* genome. The RAT-1 motif is found downstream of the *blgG* gene, which is known to be regulated by ribonucleic anti-terminators (RATs). The RAT-1 motif was found in Clostridia, Erysipelotrichia, Negativicutes, Tissierellia, Synergistetes and environmental DNA sequences. Although the consensus sequence of the RAT-1 motif shows sequence similarities to the *bglG* RAT element reported for *E. coli*, the secondary structure model of the RAT-1 motif is distinct from that of the previously reported *bglG* RAT element [21].

The PBC-6 motif. The PBC-6 motif (Additional file 9: Figure S7i**)** was initially identified in the *C. novyi* genome. There are 244 unique examples of PBC-6 motifs found in Firmicutes (mostly in Bacilli and Clostridia). The PBC-6 motif is mostly associated with genes that code for DNA-binding transcriptional regulators including the *araC* gene. Although it is possible that the PBC-6 motif might act as a protein-binding candidate at the DNA level, the run of U nucleotides present immediately after the stem suggests that this motif is likely to be a regulatory genetic element that functions at the RNA level.

The PBC-4 and PBC-2 motifs. Two transcription factor binding site candidates were discovered in the HIMB5 genome: PBC-4 (Additional file 10: Figure S8a) (308 unique examples) and PBC-2 (Additional file 10: Figure S8b) (1,200 unique examples). Transcription factor binding sites are short (typically 12-30 base-pairs), conserved palindromic inverted repeats found upstream of certain genes, and are the targets of transcription factor proteins. Binding of transcription factors to their target sites leads to regulation of the downstream genes [22]. Although we have depicted the PBC-4 and PBC-2 motifs as RNAs, the corresponding single-stranded DNAs would form the same secondary structures and might actually serve as the targets for protein binding.

The PBC-5 DNA motif. This candidate motif (Additional file 10: Figure S8c) has 98 unique examples, and is often found upstream of *rmuC*, a DNA recombination protein predicted to cleave ssDNA [23, 24]. The structure of this motif contains three hairpins with the sequence GTTTAGA appearing in the stems of the first two (orange shading). Based on the genetic context and structure, we suggest that this motif might function as a single-stranded DNA structure that is bound by the RmuC protein.

The HRC-4 motif. HRC-4 (Additional file 11: Figure S9) was first identified in *Arcobacter sp. L* species. There are 14 unique examples which are found in *Arcobacter* species and metagenomic environmental sequences. Homology searches to identify additional HRC-4 representatives resulted in the fortuitous discovery of *sig70* RNAs (described below) due largely to structural similarities between P2 of HRC-4 and P6 of the *sig70* motif. In contrast to *sig70* RNAs, HRC-4 motif representatives are not associated with a particular gene context, making it difficult to predict a biological function.

The sig70 motif. The *sig70* motif (Additional file 11: Figure S9) has 154 unique representatives and is almost exclusively found among environmental metagenomic DNA datasets. Six sequences are found in various strains of *Faecalibacterium prausnitzii* (Firmicutes) and, strangely, none are present in the original *Arcobacter sp. L* genome that gave rise to this candidate. This is an unexpected, yet very interesting outcome wherein the original IGR gave rise to the discovery of a different and much more complex motif. The original IGR from *Arcobacter sp. L* carries a three-stem junction (HRC-4 motif, Additional file 11: Figure S9), which has incidental similarities to the P6 region of the *sig70* motif. These similarities caused several hits to be initially included in the collection of HRC-4 representatives, but these were eventually recognized as a larger separate motif (*sig70*). Despite the large number of *sig70* representatives, the motif is highly conserved in primary sequence and exhibits little covariation among nucleotides forming its complex secondary structure. Approximately one third of *sig70* RNA motif sequences reside upstream of the gene for sigma 70, the RNA polymerase sigma factor that is required for initiation of RNA synthesis by combining with the core enzyme to form a holoenzyme. Other genes found downstream of the *sig70* motif are bSCRAP106 (a gene often associated with ribozymes) [25], a transcriptional regulator that binds DNA and is a member of the xenobiotic response element family, and a sigma 24 homolog (another sigma subunit involved in DNA-directed RNA polymerase). Based on genetic context, it is possible that the *sig70* motif has a role in transcription regulation by binding to the sigma 70 protein.

Supplementary References

1. Maeda H, Dudareva N. The shikimate pathway and aromatic amino acid biosynthesis in plants. Annu. Rev. Plant Biol. 2012;63:73-105.

2. Herrmann KM, Weaver LM. The shikimate pathway. Annu Rev Plant Biol. 1999;50:473-503.
3. Oxender DL, Zurawski G, Yanofsky C. Attenuation in the *Escherichia coli* tryptophan operon: role of RNA secondary structure involving the tryptophan codon region. Proc Natl Acad Sci USA. 1979;76:5524-8.
4. Neshat A, Mentz A, Rückert C, Kalinowski J. Transcriptome sequencing revealed the transcriptional organization at ribosome-mediated attenuation sites in *Corynebacterium glutamicum* and identified a novel attenuator involved in aromatic amino acid biosynthesis. J Biotechnol. 2014;190:55-63.
5. Li X, Kazan H, Lipshitz H D, Morris, QD. Finding the target sites of RNA-binding proteins. WIREs RNA. 2013;5:111-30.
6. Gottesman S, Storz G. Bacterial small RNA regulators: versatile roles and rapidly evolving variations. Cold Spring Harb Perspect Biol. 2011;3:a003796.
7. Giovannoni SJ, Bibbs L, Cho JC, Stapels MD, Desiderio R, Vergin KL, et al. Proteorhodopsin in the ubiquitous marine bacterium SAR11. Nature. 2005;438:82-5.
8. Steindler L, Schwalbach MS, Smith DP, Chan F, Giovannoni SJ. Energy starved *Candidatus Pelagibacter ubique* substitutes light-mediated ATP production for endogenous carbon respiration. PLoS ONE. 2011;6:e19725-10.
9. Winkler FK. Amt/MEP/Rh proteins conduct ammonia. Pflugers Arch. 2005;451:701-7.
10. Kouidmi I, Levesque RC, Paradis-Bleau C. The biology of Mur ligases as an antibacterial target. Mol Microbiol. 2014;94:242-53.
11. Georg J, Hess WR. *Cis*-antisense RNA, another level of gene regulation in bacteria. Microbiol Mol Biol Rev. 2011;75:286-300.
12. Sanders C, Turkarslan S, Lee D-W, Daldal F. Cytochrome c biogenesis: the Ccm system. Trends Microbiol. 2010;18:266-74.
13. Cimdins A, Roßmanith J, Langklotz S, Bandow JE, Narberhaus F. Differential control of *Salmonella* heat shock operons by structured mRNAs. Mol Microbiol. 2013;89:715-31.
14. Sowell SM, Wilhelm LJ, Norbeck AD, Lipton MS, Nicora CD, Barofsky DF, et al. Transport functions dominate the SAR11 metaproteome at low-nutrient extremes in the Sargasso Sea. ISME J. 2008;3:93-105.
15. Righetti F, Narberhaus F. How to find RNA thermometers. Front Cell Infect Microbiol. 2014;4:132.
16. Giuliodori AM, Di Pietro F, Marzi S, Masquida B, Wagner R, Romby P, et al. The *cspA* mRNA is a thermosensor that modulates translation of the cold-shock protein CspA. Mol Cell. 2010;37:21-33.
17. Keto-Timonen R, Hietala N, Palonen E, Hakakorpi A, Lindström M, Korkeala H. Cold shock proteins: a minireview with special emphasis on Csp-family of enteropathogenic *Yersinia*. Front Microbiol. 2016;7:2454-7.
18. Weinberg Z, Wang JX, Bogue J, Yang J, Corbino K, Moy RH, et al. Comparative genomics reveals 104 candidate structured RNAs from bacteria, archaea, and their metagenomes. Genome Biol. 2010;11:R31.
19. Storz G, Wolf YI, Ramamurthi KS. Small proteins can no longer be ignored. Annu Rev Biochem. 2014;83:753-77.
20. Fu Y, Deiorio-Haggar K, Anthony J, Meyer M M. Most RNAs regulating ribosomal protein biosynthesis in *Escherichia coli* are narrowly distributed to Gammaproteobacteria. Nucleic Acids Res. 2013;41:3491-503.

21. Aymerich S, Steinmetz M. Specificity determinants and structural features in the RNA target of the bacterial antiterminator proteins of the BglG/SacY family. Proc Natl Acad Sci USA. 1992;89:10410-4.
22. van Hijum SAFT, Medema MH, Kuipers OP. Mechanisms and evolution of control logic in prokaryotic transcriptional regulation. Microbiol Mol Biol Rev. 2009;73:481-509.
23. Slupska MM, Chiang JH, Luther WM, Stewart JL, Amii L, Conrad A, et al. Genes involved in the determination of the rate of inversions at short inverted repeats. Genes Cells. 2000;5:425-7.
24. Kinch LN, Ginalski K, Rychlewski L, Grishin NV. Identification of novel restriction endonuclease-like fold families among hypothetical proteins. Nucleic Acids Res. 2005;33:3598-605.
25. Weinberg Z, Kim PB, Chen TH, Li S, Harris KA, Lünse CE, et al. New classes of self-cleaving ribozymes revealed by comparative genomics analysis. Nat Chem Biol. 2015;11:606-10.
