# Supplementary figures and images for: Genome-wide discovery of structured noncoding RNAs in bacteria
Source: BMC Microbiol. 2019 Mar 22;19:66. doi: 10.1186/s12866-019-1433-7 (PMC6429828; doi:10.1186/s12866-019-1433-7)

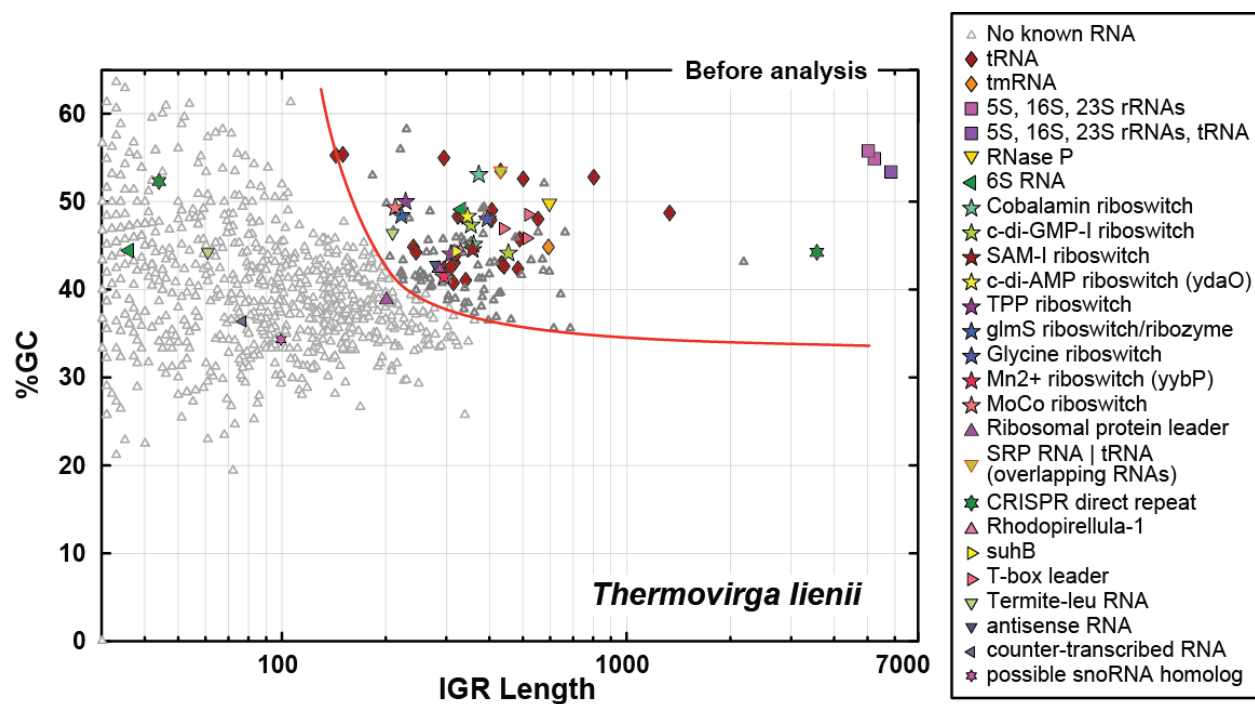

Supplement: Supplementary file 5 — Figure S3. Plots of the IGRs from the T. lienii genome sorted based on IGR length and GC content. (PDF 120 kb) [file 12866_2019_1433_MOESM5_ESM.pdf]
