# Supplementary material for: Genome-wide discovery of structured noncoding RNAs in bacteria
Source: BMC Microbiol. 2019 Mar 22;19:66. doi: 10.1186/s12866-019-1433-7 (PMC6429828; doi:10.1186/s12866-019-1433-7)
Supplement: Supplementary file 6 — Figure S4. Plots of the IGRs from the A. sp. L genome sorted based on IGR length and GC content. (PDF 95 kb) [file 12866_2019_1433_MOESM6_ESM.pdf]

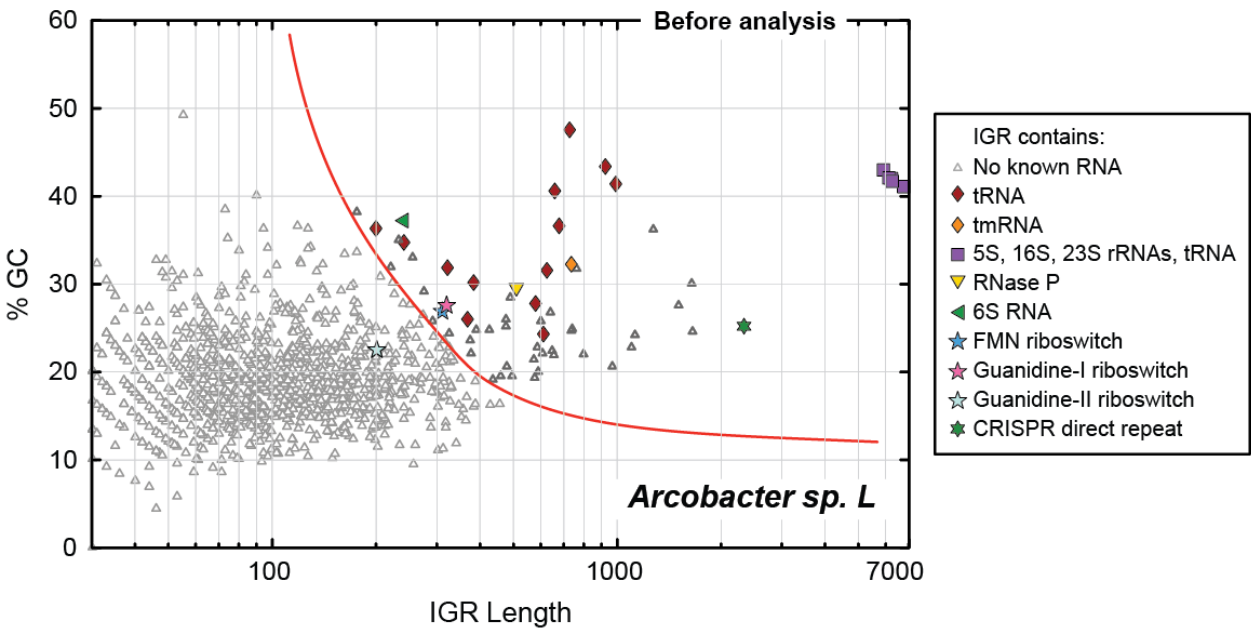

Figure S4 | Plots of the IGRs from the *A. sp. L* genome sorted based on IGR length and GC content. Details are as described in the legend to **Fig. 2a**.
