# Supplementary material for: Genome-wide discovery of structured noncoding RNAs in bacteria
Source: BMC Microbiol. 2019 Mar 22;19:66. doi: 10.1186/s12866-019-1433-7 (PMC6429828; doi:10.1186/s12866-019-1433-7)
Supplement: Supplementary file 7 — Figure S5. Plots of the IGRs from the B. cicadellinicola genome sorted based on IGR length and GC content. (PDF 66 kb) [file 12866_2019_1433_MOESM7_ESM.pdf]

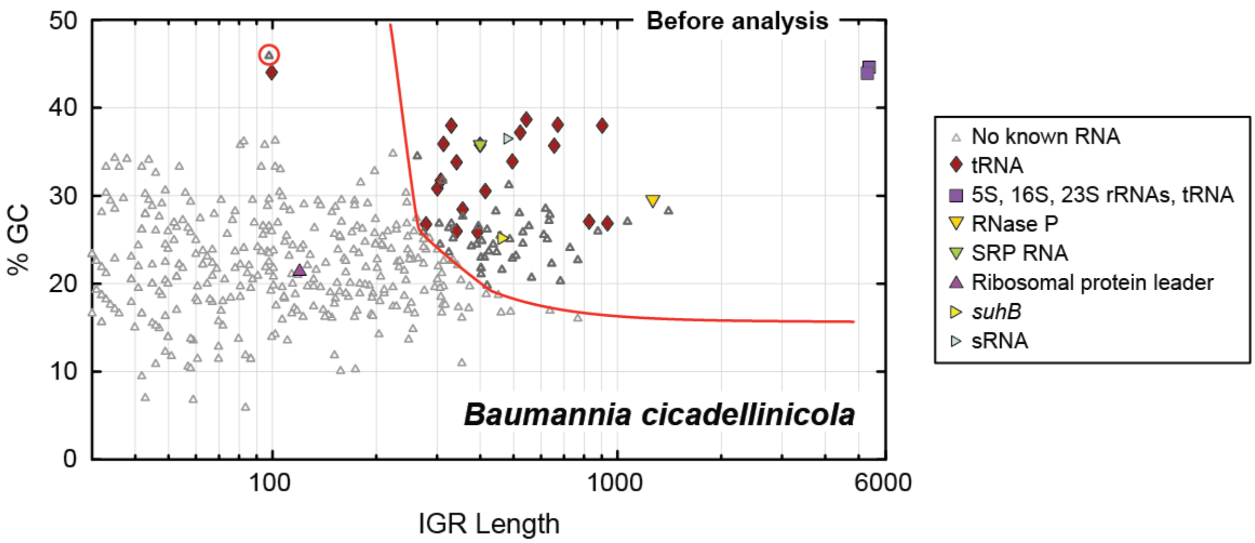

Figure S5 | Plots of the IGRs from the *B. cicadellinicola* genome sorted based on IGR length and GC content. Details are as described in the legend to **Fig. 2a**. In addition to IGRs selected with the main boundary (red line), the IGR within the red circle was also analyzed.
