# Supplementary material for: Genome-wide discovery of structured noncoding RNAs in bacteria
Source: BMC Microbiol. 2019 Mar 22;19:66. doi: 10.1186/s12866-019-1433-7 (PMC6429828; doi:10.1186/s12866-019-1433-7)
Supplement: Supplementary file 8 — Figure S6. Consensus sequence and secondary structure models for additional structured ncRNA motif candidates discovered in this study. (PDF 157 kb) [file 12866_2019_1433_MOESM8_ESM.pdf]

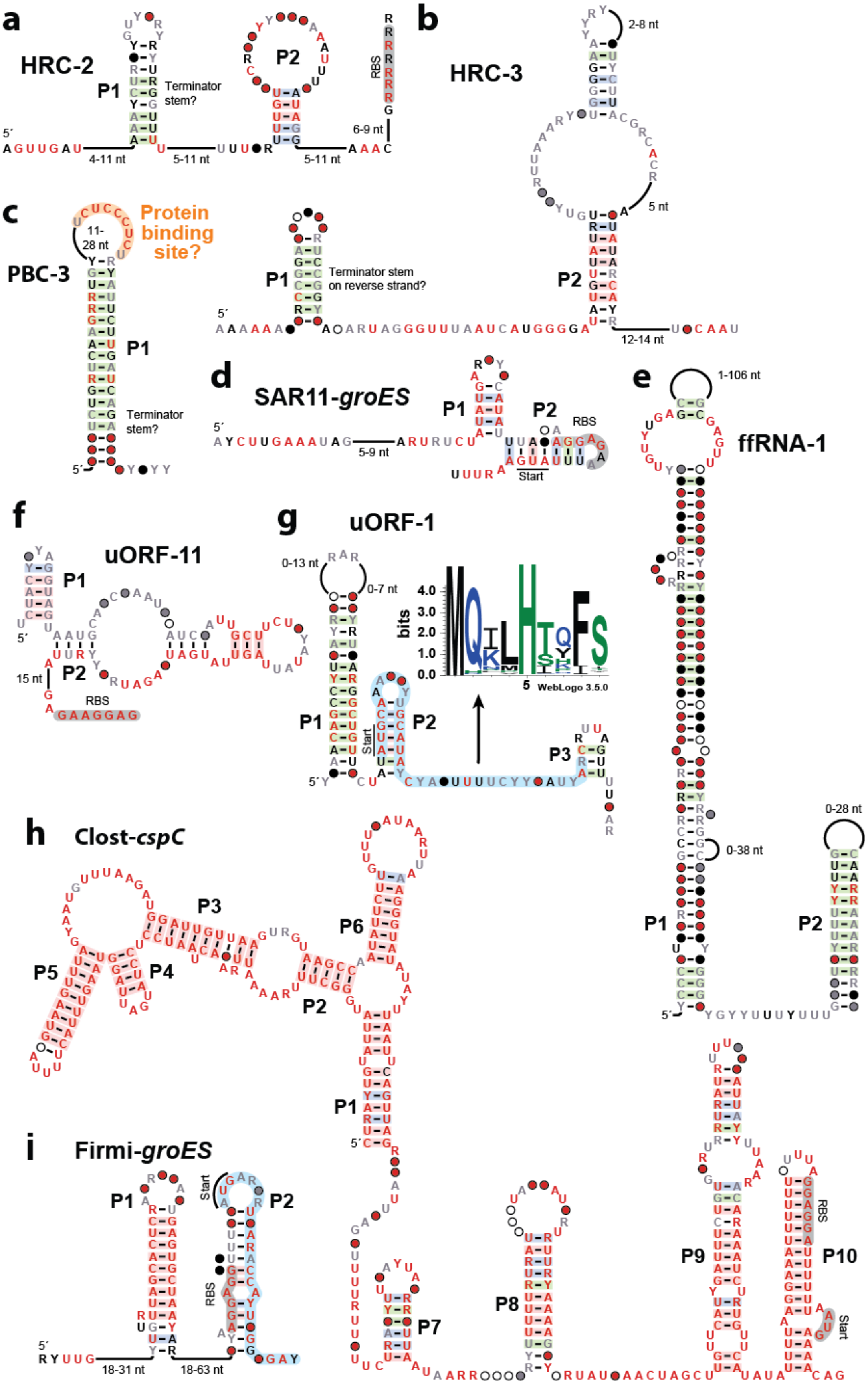
a
HRC-2
P1
P2
Terminator stem?
RBS
4-11 nt
5-11 nt
5-11 nt
6-9 nt
b
HRC-3
2-8 nt
5 nt
12-14 nt
c
PBC-3
Protein binding site?
11-28 nt
Terminator stem?
Terminator stem on reverse strand?
d
SAR11-groES
5-9 nt
RBS
Start
e
ffRNA-1
1-106 nt
0-38 nt
0-28 nt
f
uORF-11
15 nt
RBS
g
uORF-1
0-13 nt
0-7 nt
Start
P3
bits
WebLogo 3.5.0
h
Clost-cspC
P4
P5
P6
P7
P8
P9
P10
i
Firmi-groES
18-31 nt
18-63 nt

Figure S6 | Consensus sequence and secondary structure models for additional structured ncRNA motif candidates discovered in this study. Annotations are as described in the legend to **Fig. 6**. Additional details regarding each motif are presented in Additional file 1: Table S1.
