# Supplementary material for: Genome-wide discovery of structured noncoding RNAs in bacteria
Source: BMC Microbiol. 2019 Mar 22;19:66. doi: 10.1186/s12866-019-1433-7 (PMC6429828; doi:10.1186/s12866-019-1433-7)
Supplement: Supplementary file 9 — Figure S7. Consensus sequences and secondary structure models for additional ncRNA motif candidates or candidates with other functions discovered in this study. (PDF 385 kb) [file 12866_2019_1433_MOESM9_ESM.pdf]

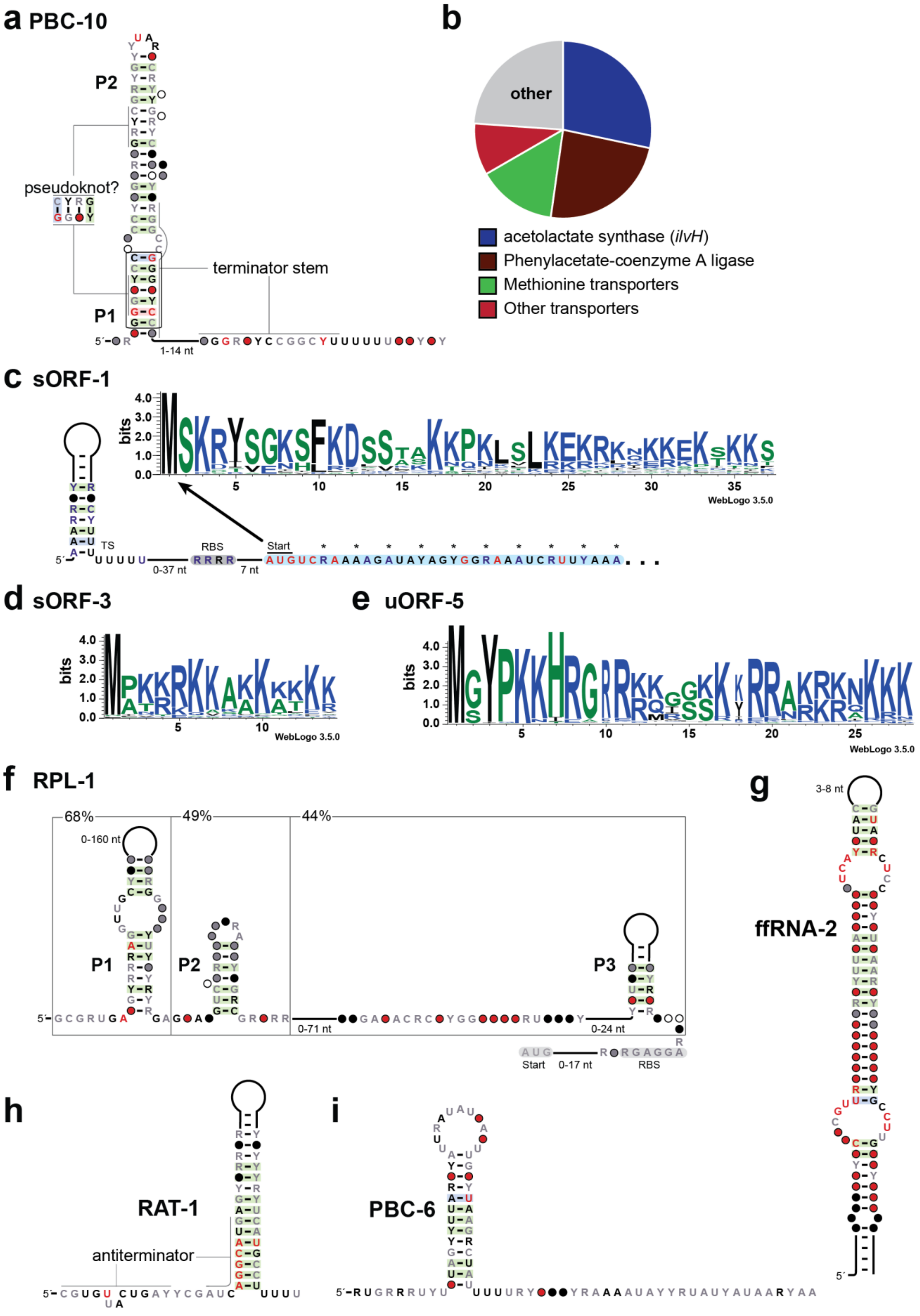
a PBC-10
P2
pseudoknot?
terminator stem
P1
1-14 nt
b
other
acetolactate synthase (ilvH)
Phenylacetate-coenzyme A ligase
Methionine transporters
Other transporters
c sORF-1
bits
WebLogo 3.5.0
TS
RBS
Start
0-37 nt
7 nt
d sORF-3
e uORF-5
f RPL-1
68%
49%
44%
0-160 nt
P1
P2
P3
0-71 nt
0-24 nt
Start
0-17 nt
RBS
g
3-8 nt
ffRNA-2
h
RAT-1
antiterminator
i
PBC-6

Figure S7 | Consensus sequences and secondary structure models for additional ncRNA motif candidates or candidates with other functions discovered in this study. a, Sequence and secondary structure model for the PBC-10 motif RNA. Annotations are as described for **Fig. 3**. Palindromic sequence near the origin of the P1 stem is boxed. **b,** Genes associated with the PBC-10 motif RNA. **c-e,** Candidates containing ORFs. Annotations are as described in the legend to **Fig. 6**. **f-i** Sequence and secondary structure model for candidates with other functions. Details regarding each motif are presented in Additional file 1: Table S1.
