# Supplementary material for: Genome-wide discovery of structured noncoding RNAs in bacteria
Source: BMC Microbiol. 2019 Mar 22;19:66. doi: 10.1186/s12866-019-1433-7 (PMC6429828; doi:10.1186/s12866-019-1433-7)
Supplement: Supplementary file 10 — Figure S8. Consensus sequence and secondary structure models for small ncRNA motif candidates and a candidate ssDNA motif. (PDF 69 kb) [file 12866_2019_1433_MOESM10_ESM.pdf]

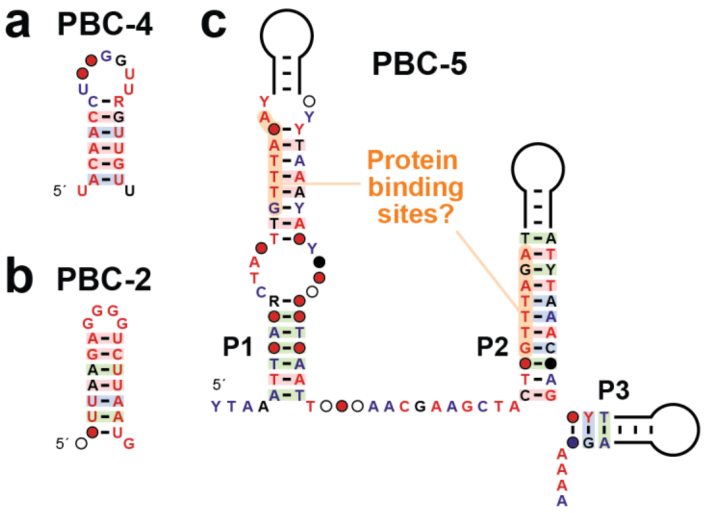

Figure S8 | Consensus sequence and secondary structure models for small ncRNA motif candidates and a candidate ssDNA motif. a, b, Small motifs presumed to function as RNA binding sites for protein factors. **c,** Possible structured single-stranded DNA motif that is bound by protein. Annotations are as described in the legend to **Fig. 6**. Details regarding each motif are presented in Additional file 1: Table S1.
