# Supplementary material for: Genome-wide discovery of structured noncoding RNAs in bacteria
Source: BMC Microbiol. 2019 Mar 22;19:66. doi: 10.1186/s12866-019-1433-7 (PMC6429828; doi:10.1186/s12866-019-1433-7)
Supplement: Supplementary file 11 — Figure S9. Fortuitous discovery of a large structured motif. (PDF 136 kb) [file 12866_2019_1433_MOESM11_ESM.pdf]

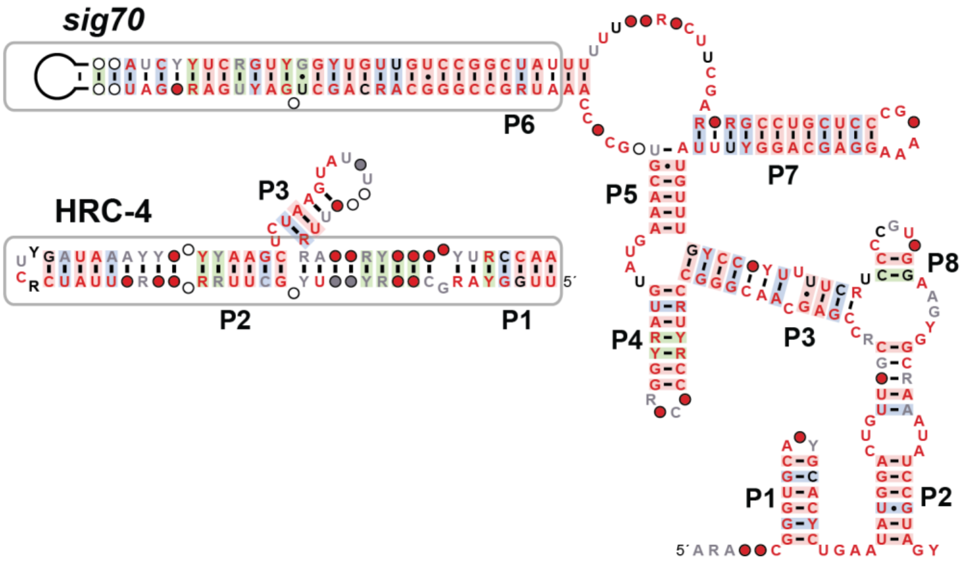

Figure S9 | Fortuitous discovery of a large structured motif. Comparison of the vague similarities between the consensus sequence and secondary structure models for the HRC-4 motif first identified from *Arcobacter sp. L*, and the *sig70* motif. The gray boxes identify the regions of the two RNAs that are vaguely similar, which caused representatives of the *sig70* motif to be included in the collection of bioinformatics hits for HRC-4. After further analysis, we separated the RNA representatives into two distinct classes, and discovered many additional examples of the *sig70* motif. Note that the *sig70* motif is not found among any of the five genomes analyzed in detail in the current study.
